# Targeting the KCNC3‐Dependent Anterior Insular‐Lateral Orbitofrontal Cortex Glutamatergic Circuit Attenuates Post‐Traumatic Anxiety

**DOI:** 10.1002/advs.76779

**Published:** 2026-07-27

**Authors:** Meng‐Ge Li, Xiao‐Bo Qian, Hai‐Long Zhang, Di Li, Ling Ji, Xin‐Chun Xu, Jia‐Sheng Ding, Li‐Jun Yin, Li Zhang, Guang‐Yin Xu, Rong Gao

**Affiliations:** ^1^ Center of Translational Medicine Department of Neurosurgery Department of Medical Imaging The Zhangjiagang Affiliated Hospital of Soochow University Zhangjiagang Jiangsu China; ^2^ Center of Translational Medicine The Fourth Affiliated Hospital of Soochow University Suzhou China; ^3^ Department of Neurosurgery & Brain and Nerve Research Laboratory The First Affiliated Hospital of Soochow University Suzhou China; ^4^ Jiangsu Key Laboratory of Neuropsychiatric Diseases and Translational Research for Brain Diseases Institute of Neuroscience Soochow University Suzhou China

**Keywords:** anterior insular cortex, KCNC3, lateral orbitofrontal cortex, post‐traumatic anxiety, traumatic brain injury

## Abstract

Traumatic brain injury (TBI) is a prevalent cause of secondary neuropsychiatric disorders, with post‐traumatic anxiety significantly worsening long‐term outcomes. However, the key neural circuits and molecular mechanisms underlying post‐TBI anxiety remain poorly understood. Here, we show that TBI patients with frontal lobe injuries and anxiety exhibited increased activity and enhanced functional connectivity in the anterior insular cortex (aIC) and lateral orbitofrontal cortex (lOFC). Mechanistically, we established a mouse model of moderate frontal lobe TBI, categorizing animals into anxious and non‐anxious TBI groups. BOLD‐fMRI and c‐Fos expression analyses revealed that mice with post‐traumatic anxiety also exhibited increased activity in the aIC and lOFC, as well as heightened activity in the aIC‐lOFC glutamatergic circuit. Optogenetic and chemogenetic modulation of this circuit bidirectionally regulated post‐traumatic anxiety. Single‐cell RNA sequencing of aIC glutamatergic neurons identified a specific downregulation of KCNC3 in anxious TBI mice. Functional validation confirmed that KCNC3 downregulation mediates hyperactivity in the aIC‐lOFC glutamatergic circuit and induces post‐traumatic anxiety. Together, these findings unveil a critical role of TBI‑induced KCNC3 downregulation in aIC glutamatergic neurons in driving hyperactivity of the aIC–lOFC glutamatergic circuit and resulting post‑traumatic anxiety, and they suggest KCNC3 and the aIC–lOFC circuit as promising therapeutic targets for post‑TBI anxiety.

## Graphical abstract

Frontal lobe traumatic brain injury is associated with hyperactivity of an insular–orbitofrontal circuit in both patients and mice. By combination of integrating functional imaging, cell‐type–specific circuit manipulation, single‐cell transcriptomics, and whole‐cell recordings, this work identifies the downregulation of the potassium channel KCNC3 in anterior insular glutamatergic neurons as a key driver of post‐traumatic anxiety and a promising therapeutic entry point.

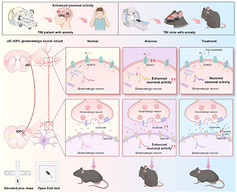

## 1 Introduction

Traumatic brain injury (TBI) triggers a cascade of complex secondary sequelae, including somatic symptoms, cognitive impairments, and emotional disturbances [1, 2, 3]. Notably, anxiety, depression, and cognitive dysfunction are particularly pronounced, with studies indicating that TBI patients face several‐fold increased relative risk of developing anxiety disorders compared to the general population [4, 5, 6]. Approximately 20%–36% of TBI patients develop anxiety disorders [7, 8, 9, 10, 11, 12]. Anxiety is recognized as one of the most prevalent and earliest psychiatric manifestations after TBI [13, 14, 15]. Longitudinal observations indicate that, over a ten‐year period, anxiety symptoms in TBI patients may fluctuate, with periods of both exacerbation and remission; nevertheless, these symptoms rarely decrease to levels classified as mild or lower [16]. Traditional anxiolytic medications demonstrate limited effectiveness in managing anxiety in TBI patients. Therefore, elucidating the neural circuits and molecular mechanisms underlying anxiety disorders after TBI is crucial for understanding the pathophysiology of these conditions and for developing effective therapeutic strategies.

Converging evidence highlights neural circuit research as central to understanding the mechanisms underlying pathological anxiety [17, 18]. Abnormalities in regions such as the OFC and IC have been shown to trigger anxiety, primarily through disruption of frontal‐limbic functional connectivity [19, 20, 21]. Functional imaging and clinical studies consistently demonstrate that the insular cortex is critical for the generation and perception of negative emotions, including anxiety and fear. Similarly, the orbitofrontal cortex is central to emotional regulation and decision‐making [22, 23]. Notably, patients with anxiety disorders exhibit reduced volume or impaired activation in both the insular and orbitofrontal cortex [24, 25, 26]. However, the precise role and molecular mechanisms of the neural circuit between the aIC and lOFC in anxiety following TBI remain unclear. Despite these insights, research on the neural circuit and molecular mechanisms of anxiety resulting from frontal lobe injury remains limited, with a lack of established neuroimaging and molecular biomarkers. Thus, further investigation into anxiety following frontal lobe damage is warranted, as this may reveal distinct pathophysiological pathways and identify new targets for diagnosis and treatment.

Post‐traumatic anxiety affects about 30% of TBI survivors, persists for decades, and is more refractory to remission than depression; yet no pharmacological intervention carries level‐I evidence in this population. SSRIs show modest effect sizes, and benzodiazepines are contra‐indicated because of cognitive and gait side effects. The absence of (i) circuit‐based mechanistic insight that explains inter‐individual variability, (ii) predictive biomarkers for patient stratification, and (iii) druggable targets that can be engaged safely after acute brain injury collectively represent a major translational bottleneck. Identifying a cross‐species conserved circuit that both mediates anxiety and is amenable to selective modulation therefore constitutes an urgent unmet need in neurotrauma care.

*KCNC3* gene encodes the KCNC3 potassium channel subunit, which regulating neuronal excitability and action potential firing patterns in fast‐firing neurons. Abnormal function of the KCNC3 channel can lead to dysregulation of the excitatory‐inhibitory balance in neurons [27, 28]. Notably, the KCNC3 channel also facilitates the endocytosis of synaptic vesicles by organizing the F‐actin cytoskeleton at nerve terminals, thereby playing a specific role in neural circuit remodeling and dysfunction following TBI [29, 30]. Genetic variations in KCNC3 play a critical role in neurological disorders. Missense mutations in KCNC3 result in a complete loss of voltage‐gated conductance, broaden action potentials, reduce neuronal firing, and induce learning deficits in mice [31]. Deletion of KCNC3 increased presynaptic action potential duration and facilitated excitatory transmitter release [27]. Although the involvement of KCNC3 in multiple psychiatric disorders has been demonstrated, its mechanism of action in anxiety disorders has not been fully elucidated.

In the study, we focused on patients with frontal lobe injury and anxiety disorders. Through telephone follow‐ups, Glasgow Coma Scale (GCS) scores, and Hamilton Rating Scale (HAMA) for Anxiety scores [32], we screened patients with frontal lobe injury and anxiety disorders for the experimental group and those with frontal lobe injury without anxiety disorders for the control group. BOLD‐fMRI imaging confirmed significantly increased activity in the IC and OFC of patients and mice with post‐traumatic anxiety. This study elucidated the critical role of the aIC‐lOFC glutamatergic circuit in post‐traumatic anxiety and identified KCNC3 as a key molecule mediating the abnormal activity of this circuit. These findings enhance our understanding of the neural mechanisms underlying post‐traumatic anxiety, identifying potential targets for the development of novel therapeutic strategies.

## 2 Results

### 2.1 Increased Activity and Enhanced IC‐OFC Functional Connectivity in TBI Patients With Anxiety

**FIGURE 1:**
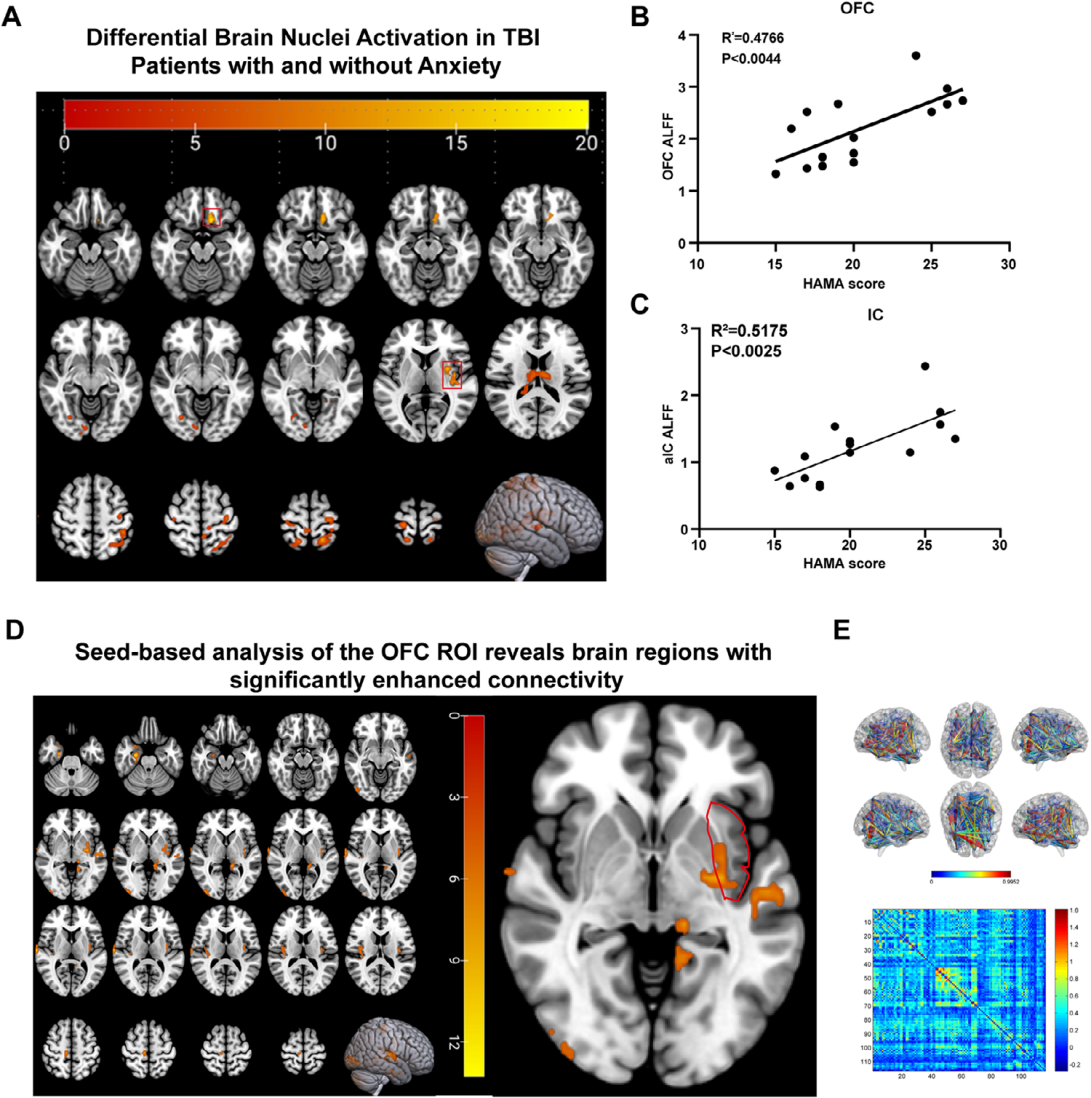
Distinct neural activation patterns in TBI patients with comorbid anxiety disorders. (A) Whole‐brain activation maps (voxel‐level *p* < 0.001, cluster‐level *p* < 0.05, FWE‐corrected, 95% CI [‐0.5161, 0.2495]) revealed hyperactivation in anxious TBI patients (n = 15) compared to non‐anxious TBI (n = 15), with peak differences localized to the IC, OFC, and precentral gyrus. (B, C) HAMA scores exhibited a robust positive correlation with IC and OFC activity (OFC: R^2^ = 0.4766, *p <* 0.0044, Pearson's correlation, 95% CI [0.2755, 0.8884] and IC: R^2^ = 0.5175, p < 0.0025, Pearson's correlation 95% CI [0.5340, 0.9387]). (D) Seed‐based functional connectivity analysis using the OFC as the ROI (voxel *p* < 0.001, cluster *p* < 0.05, FWE‐corrected, 95% CI [‐1.149, 0.08960]) demonstrated enhanced coupling between the OFC and key limbic and associative regions, including the IC, Precuneus‐L, Putamen‐L, Temporal‐Sup‐R, and precentral‐L, with the IC showing the strongest connectivity increase. (E) Whole‑brain functional connectivity matrices (threshold at voxel *p* < 0.001, cluster *p* < 0.05, FWE‐corrected, 95% CI [‐0.1478, 0.1464]) revealed widespread hyperconnectivity in anxious TBI patients, particularly within the salience network and default mode network.

Traumatic brain injury (TBI) patients with frontal lobe damage showed a heightened risk of developing anxiety disorders in our preliminary observations. Using Glasgow Coma Scale (GCS) scores, structured telephone follow‑ups, and Hamilton Anxiety Rating Scale (HAMA) assessments, we identified TBI patients with and without anxiety and acquired BOLD‑fMRI scans. To probe the central neural mechanisms underlying this heightened vulnerability, we evaluated the dynamics of the amplitude of low‑frequency fluctuations (ALFF), an index of regional spontaneous brain activity [33, 34]. Whole‐brain analysis revealed significantly increased ALFF dynamics in multiple brain regions in TBI patients with anxiety, including OFC, IC, Temporal Sup L, ParaHippocampal‐R and Thalamus‐R (Figure 1A and Figure S1A). Correlation analysis between altered brain regions and HAMA scores demonstrated that higher HAMA scores were strongly associated with increased OFC and IC activity, indicating a significant relationship between OFC and IC activity with anxiety severity in TBI patients (Figure 1B,C). These findings support a robust link between OFC and IC activity and the severity of anxiety in post‐traumatic anxiety.

Subsequent seed‐based region‐of‐interest (ROI) analysis using the OFC as the seed revealed significantly enhanced connectivity from the IC, Precuneus‐L, Putamen‐L, Temporal‐Sup‐R, and precentral‐L to the OFC (Figure 1D and Figure S1B). These results suggest that the IC‐OFC circuit may play a crucial role in the anxiety following TBI. Using the AAL116 atlas, we extracted mean BOLD signals from each brain region, calculated Pearson correlation coefficients between regions, and performed Fisher‐Z transformations. A two‐sample *t*‐test revealed significant differences in brain connectivity between groups, with a significance level of 0.001. Multiple comparison corrections, including Bonferroni, FWE, and network‐based statistic (NBS), identified 50 different connections (Figure 1E).

### 2.2 Frontal Lobe Injury Induces Activity in the aIC and lOFC of Anxious TBI Mice

**FIGURE 2:**
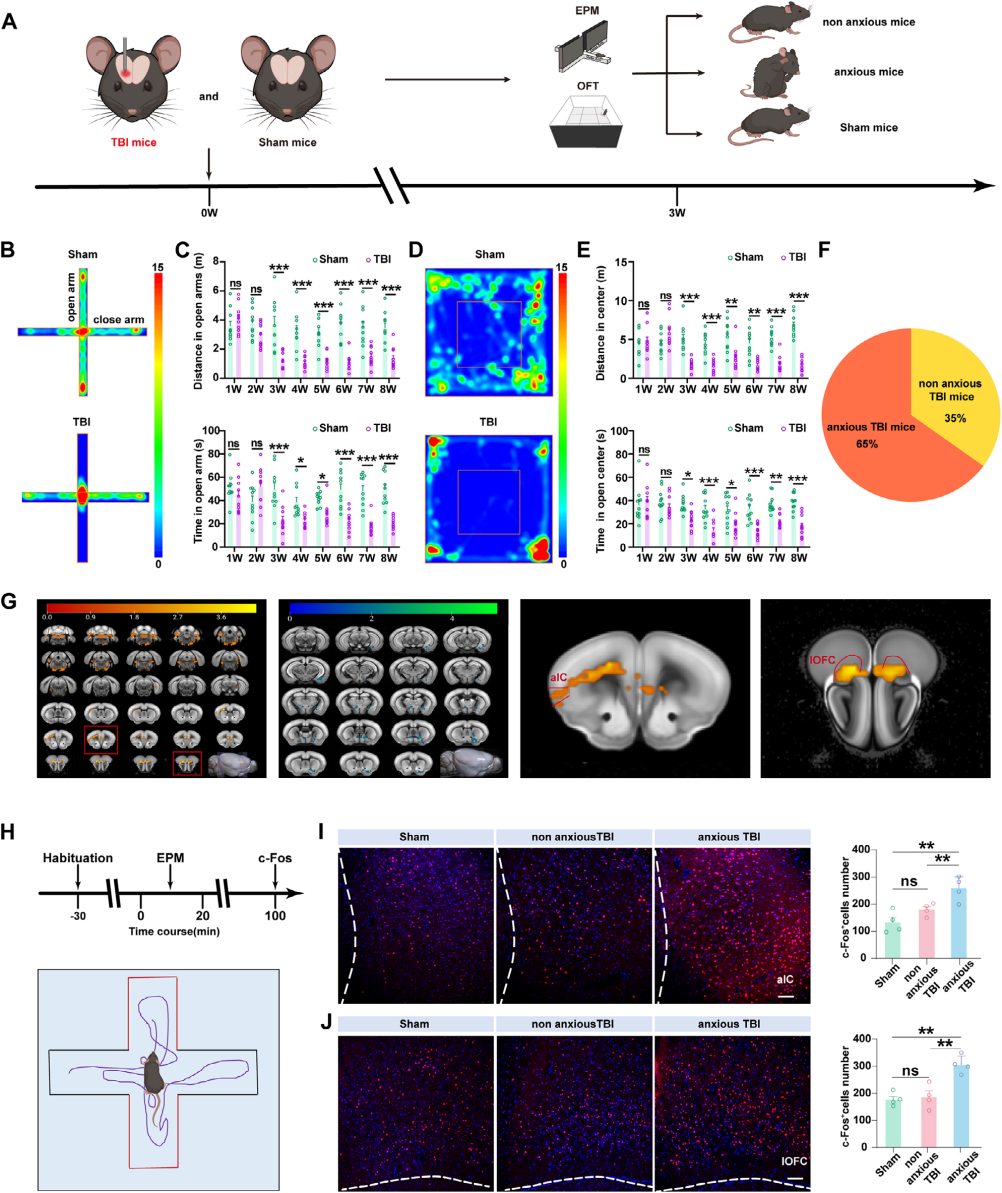
Enhanced activity of aIC and lOFC in frontal TBI‐induced anxiety mice. (A) Schematic of mice behavioral paradigm. (B, D) Heatmaps depicting spatial exploration patterns in the EPM (B) and OFT (D). (C, E) Quantitative analyses of distance traveled and time spent in the open arms of the EPM (C) and the central area of the OFT (E). (F) Pie chart summarizing the percentage distribution of anxious vs. non‐anxious phenotypes among frontal TBI mice. (G) Coordinate maps showing significantly hyperexcitable and hypoexcitable regions identified by amplitude of ALFF analysis of BOLD‐fMRI in anxious TBI mice compared to non‐anxious TBI mice (voxel‐level p < 0.001, cluster‐level p < 0.05, FWE‐corrected G, n = 10 per group). (H) Experimental workflow illustrating the procedures for c‐Fos immunofluorescence labeling following EPM exposure. (I, J) Representative immunofluorescence images of c‐Fos‐positive cells in the aIC (I) and lOFC (J) in Sham, non‐anxious TBI, and anxious TBI mice (scale bar, 100 µm), and corresponding quantitative analyses of c‐Fos‐positive cells. Data are presented as mean ± SEM. Statistical analysis was performed using two‐way ANOVA followed by Sidak's multiple comparisons test, C, E, n = 11 per group); one‐way ANOVA followed by Tukey's multiple comparisons test, I, J, n = 4 per group); ns = not significant, ^*^ *p* < 0.05, ^**^ *p* < 0.01, ^***^ *p* < 0.001.

Clinical data indicate a high prevalence of anxiety disorders in patients with frontal lobe injuries. Based on these observations, we employed the controlled cortical impact (CCI) model to induce a moderate frontal lobe injury model in mice (Figure S2A). The elevated plus maze (EPM) and open field test (OFT) were used to assess anxiety‐like behaviors (Figure 2A). TBI male mice spent less time and traveled shorter distances in the open arms of the EPM (Figure 2B,C) and in the center of the OFT (Figure 2D,E), indicative of anxiety‐like behaviors. Importantly, TBI mice did not exhibit motor deficits (Figure S2B,C). Anxiety‐like behaviors emerged during the third week post‐injury and persisted through the eighth week, indicating a sustained anxiety‐like phenotype lasting up to 8 weeks after injury (Figure 2B–E). Statistical analysis showed that 65% of TBI male mice exhibited significant anxiety‐like behaviors (Figure 2F). To investigate the mechanisms underlying this variability, we categorized TBI mice into two groups: anxious TBI and non‐anxious TBI, resulting in three experimental groups: Sham, non‐anxious TBI, and anxious TBI.

To investigate the neural circuitry underlying post‐TBI anxiety, we performed 9.4 T BOLD‐fMRI on anxious and non‐anxious TBI mice. ALFF analysis revealed significantly increased activity in the orbitofrontal cortex (lOFC), anterior insular cortex (aIC), prefrontal cortex, anterior cingulate cortex, S1, and thalamus in anxious TBI mice compared to non‐anxious mice. The nucleus accumbens, preoptic nucleus of the thalamus, and caudate nucleus (Figure 2G). Consistently, EPM exposure followed by perfusion and c‐Fos immunofluorescence labeling showed a marked increase in c‐Fos‐positive neurons in the lOFC and aIC of anxious TBI mice, whereas Sham and non‐anxious TBI mice showed no significant differences (Figure 2H–J). These results are in line with the clinical BOLD‐fMRI data from TBI patients with anxiety and suggest that the aIC and lOFC constitute key nodes mediating post‐traumatic anxiety.

### 2.3 AIC Glutamatergic Neurons Critically Mediate Anxiety‐Like Behaviors in Anxious TBI Mice

**FIGURE 3:**
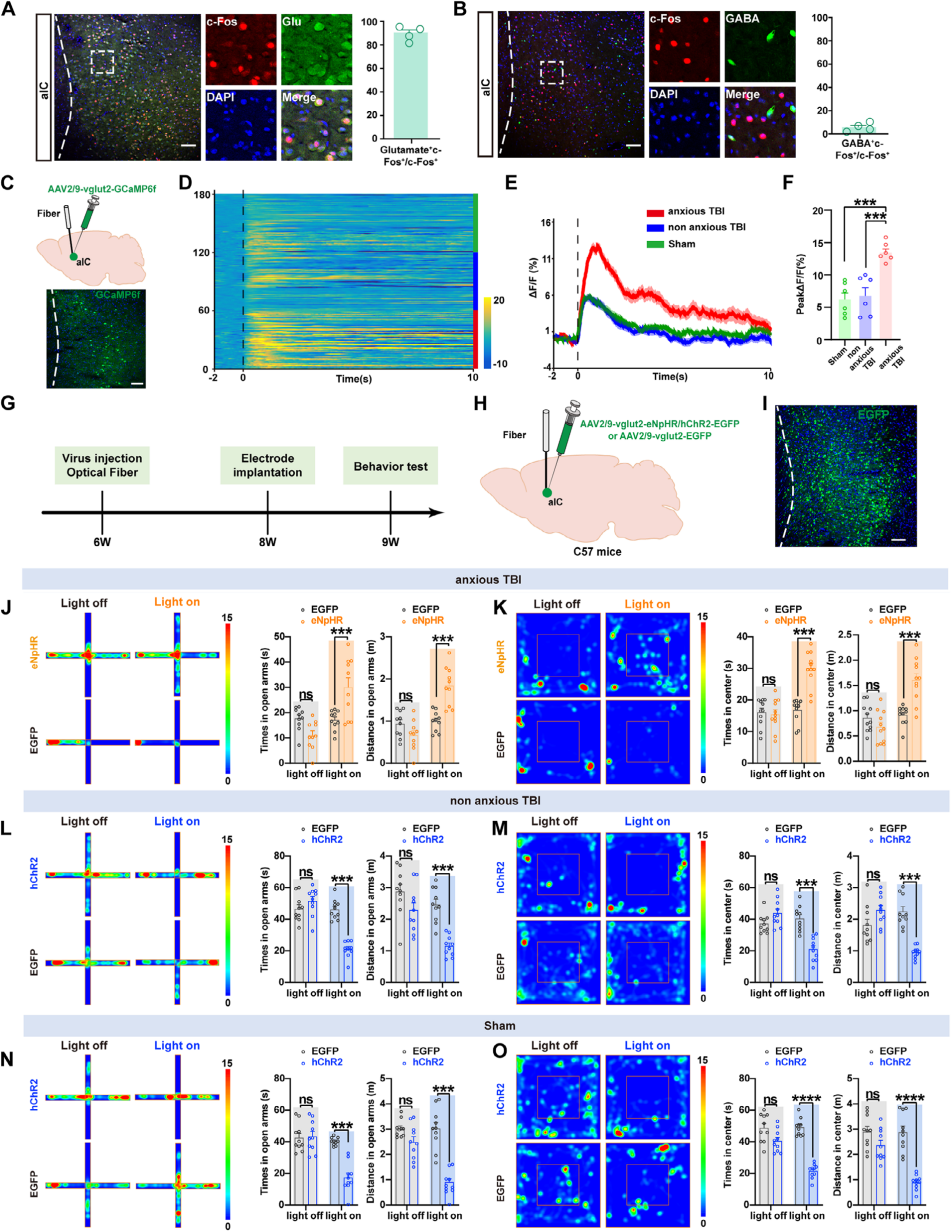
Critical role of aIC glutamatergic neurons in anxiety behavioral responses in anxious TBI mice. (A, B) llustration and quantitative analysis of c‐Fos–positive cells co‐labeled with glutamatergic (A) or GABAergic (B) neurons in the aIC (scale bar,100 µm), statistical analysis of co‐localization between c‐Fos‐positive cells with glutamatergic (A) or GABA (B) neurons in the aIC. (C) Diagram of viral injection sites and representative fluorescence expression in the aIC (scale bar, 50 µm). (D–F) Heatmaps (D) and signal traces (E) of calcium signals in aIC glutamatergic neurons from Sham, non‐anxious TBI, and anxious TBI mice, statistical analysis of peak calcium signal activity in aIC glutamatergic neurons (F). (G–I) Schematic of the optogenetic manipulation workflow (G), diagram of viral injection sites (H), and representative expression in the aIC (I); scale bar: 50 µm). (J–O) Representative heatmaps and quantification of EPM and OFT performance under eNpHR stimulation in anxious TBI mice (J, K) or hChR2 stimulation in non‐anxious TBI (L, M) and Sham mice (N, O). EGFP‐expressing mice served as negative controls to minimize potential confounding effects of repeated experimental exposure. Data are presented as mean ± SEM. Statistical analysis was performed using one‐way ANOVA followed by Tukey's multiple comparisons test, F, n = 6 per group); two‐way ANOVA followed by Sidak's multiple comparisons test, (J–O), n = 10–11 per group); ns = not significant, ^***^ *p* < 0.001.

To further identify the neurons in the aIC associated with anxiety‐like behaviors, we used markers for glutamatergic and GABAergic neurons. Following EPM exposure, the majority of c‐Fos–positive cells in the aIC were glutamatergic neurons (Figure 3A,B). In vivo fiber photometry experiments showed that, during exploratory behavior in the open arms of the EPM, robust calcium signals were recorded from aIC glutamatergic neurons. Notably, anxious TBI mice displayed significantly elevated calcium activity in aIC glutamatergic neurons, as reflected by increased peak calcium responses (Figure 3C–F). By contrast, no significant differences in calcium activity were observed in the aIC glutamatergic neurons of Sham and non‐anxious TBI mice (Figure 3C–F). These results demonstrate that anxious TBI mice exhibit significantly enhanced calcium activity in aIC glutamatergic neurons.

To directly test the causal role of aIC glutamatergic neurons in anxiety‐like behaviors, we combined optogenetic manipulations with EPM and OFT paradigms (Figure 3G–I). We injected light‐sensitive or control viral vectors specifically targeting glutamatergic neurons in the aIC. Application of 589 nm yellow light to activate eNpHR and inhibit aIC glutamatergic neurons significantly alleviated anxiety‐like behaviors in anxious TBI mice (Figure 3J,K). Conversely, activation of aIC glutamatergic neurons with 473 nm blue light increased anxiety‐like behaviors in Sham and non‐anxious TBI mice (Figure 3L‐O). In contrast, neither inhibition nor activation of cEGFP‐expressing control neurons in the aIC altered anxiety‐like behaviors in anxious TBI, non‐anxious TBI and Sham mice (Figure 3J–O). Collectively, these findings underscore the pivotal role of aIC glutamatergic neurons in modulating anxiety‐like behaviors in TBI mice.

### 2.4 LOFC Glutamatergic Neurons Drive Anxiety‐Like Behaviors in TBI Mice

**FIGURE 4:**
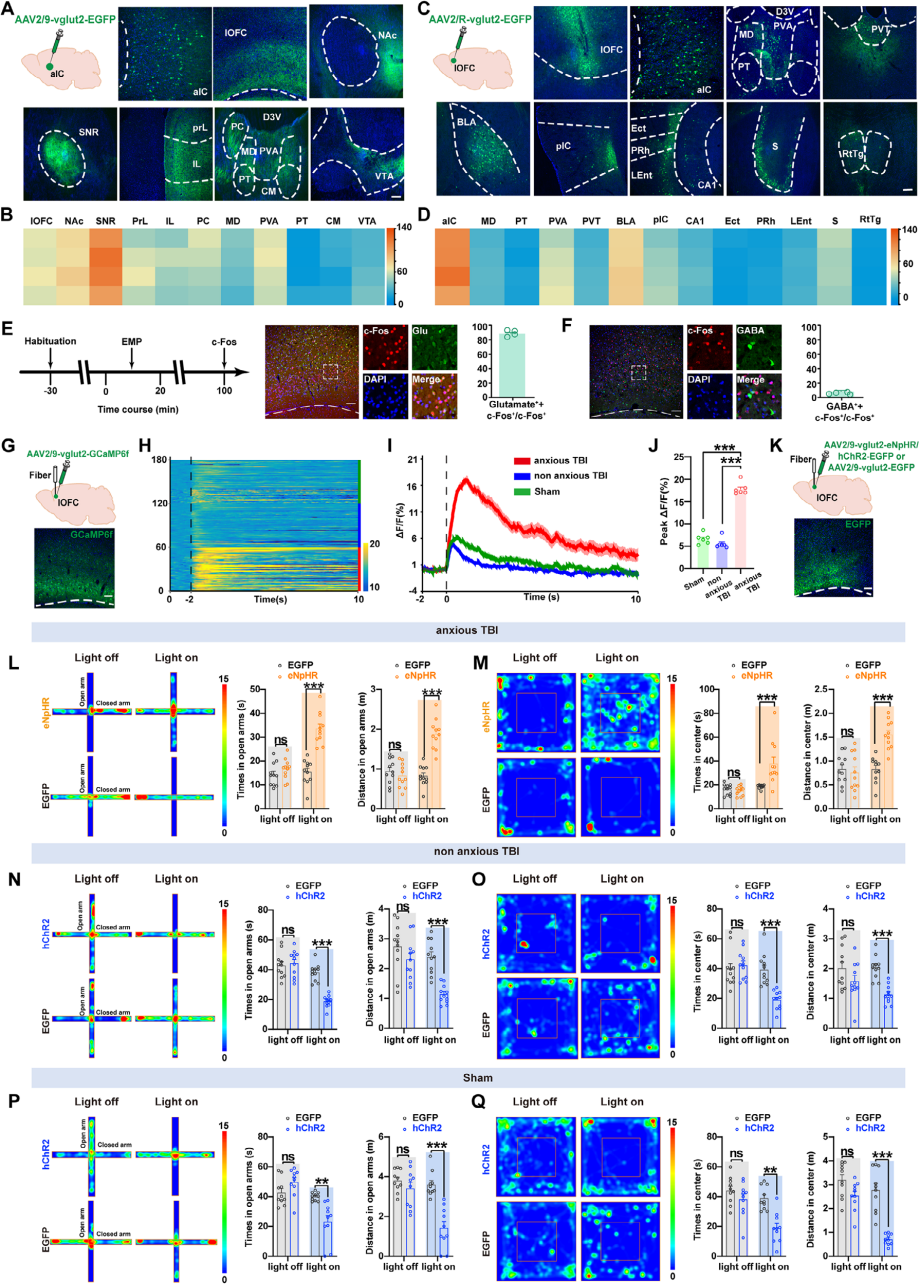
Critical role of lOFC glutamatergic neurons in anxiety behavioral responses in anxious TBI mice. (A, B) Diagram of viral injection sites and viral expression within the aIC, viral‐labeled terminals and fluorescence intensity heatmap of the downstream brain (scale bar, 100 µm). (C, D) Diagram of viral injection sites and viral expression in the lOFC, viral expression and fluorescence intensity heatmap of the upstream brain regions of the lOFC (scale bar, 100 µm). (E, F) llustration and quantitative analysis of c‐Fos–positive cells co‐labeled with glutamatergic (E) or GABAergic (F) neurons in the lOFC. (scale bar, 100 µm). (H) Heatmaps of calcium signals in lOFC glutamatergic neurons from Sham, non‐anxious TBI, and anxious TBI mice. (I) Calcium signal traces of lOFC glutamatergic neurons in Sham, non‐anxious TBI, and anxious TBI mice. (J) Statistical analysis of peak calcium signal activity in lOFC glutamatergic neurons. (K) Schematic of viral injection sites and representative expression in the aIC (K; scale bar: 50 µm). (L–Q) Representative heatmaps and quantification of EPM and OFT performance under eNpHR stimulation in anxious TBI mice (L, M) or hChR2 stimulation in non‐anxious TBI (N, O) and Sham mice (P, Q). EGFP‐expressing mice served as negative controls to minimize potential confounding effects of repeated experimental exposure. Data are presented as mean ± SEM. Statistical analysis was performed using one‐way ANOVA followed by Tukey's multiple comparisons test, n = 6 per group, (J); two‐way ANOVA followed by Sidak's multiple comparisons test, n = 10–11 per group, (L–O); ns = not significant, ^**^ *p* < 0.01, ^***^ *p* < 0.001.

Previous studies demonstrated significantly heightened calcium signaling activity in glutamatergic neurons within the aIC of mice exhibiting post‐traumatic anxiety. To elucidate the neuroanatomical connectivity between aIC and the lOFC, anterograde tracing viruses were injected into the aIC (Figure 4A). We discovered that aIC^Glu^ project to the lOFC, nucleus accumbens (NAc), substantia nigra reticulata (SNR), prelimbic cortex (PrL), infralimbic cortex (IL), parietal cortex (PC), mediodorsal thalamus (MD), paraventricular nucleus of the thalamus (PVA), central medial thalamus (CM), and ventral tegmental area (VTA), with particularly strong projections to the lOFC (Figure 4A,B). Additionally, we injected retrograde viruses into the lOFC to identify the brain regions that project to it. The results indicated that the aIC, MD, posterior thalamus (PT), PVA, paraventricular nucleus of the thalamus (PVT), basolateral amygdala (BLA), posterior insular cortex (pIC), entorhinal cortex (Ect), perirhinal cortex (PRh), lateral entorhinal cortex (LEnt), CA1, and rostral thalamic group (RtTg) all project to the lOFC (Figure 4C). Immunofluorescence staining revealed that the GFP expression within the aIC region predominantly colocalized with glutamatergic neurons (Figure S3A). To further confirm the aIC‐ IOFC projection, an anterograde monosynaptic tracking strategy was performed by injecting an AAV2/1‐vglut2‐Cre virus into the aIC and an AAV2/9‐EF1α‐mCherry virus into the IOFC. The mCherry^+^ neurons were observed in the IOFC 3 weeks later, which were largely stained positively with a glutamate antibody (Figure S3B). These findings indicate that glutamatergic neurons in the aIC monosynaptically project to glutamatergic neurons in the lOFC, providing an anatomical foundation for understanding the neural circuit mechanisms underlying anxiety‐like behaviors following TBI.

To further identify neurons in the lOFC associated with anxiety behavior, we examined markers for glutamatergic and GABAergic neurons. Immunostaining revealed that EPM‐induced c‐Fos‐positive cells in the lOFC predominantly colocalized with glutamatergic rather than GABAergic neurons (Figure 4E,F). Fiber photometry during EPM exposure demonstrated heightened calcium activity in lOFC glutamatergic neurons of anxious TBI mice, evidenced by increased peak amplitude during open‐arm exploration (Figure 4G–J). No significant differences were observed in the calcium activity of lOFC glutamatergic neurons in Sham and non‐anxious TBI mice (Figure 4J). These results demonstrate that anxious TBI mice exhibit significantly enhanced calcium activity in aIC glutamatergic neurons.

To further explore the regulatory role of lOFC glutamatergic neurons in anxiety‐like behaviors, we employed optogenetic manipulation and assessed behaviors using EPM and OFT (Figure 4K–Q). Injection of light‐sensitive and control viral‐infected lOFC glutamatergic neurons, followed by 589 nm yellow light application, inhibited these neurons and alleviated anxiety‐like behaviors in anxious TBI mice (Figure 4L,M). Conversely, 473 nm blue light activation of lOFC glutamatergic neurons increased anxiety‐like behaviors in Sham and non‐anxious TBI mice (Figure 4N‐Q). However, neither inhibition nor activation of control viral‐infected aIC glutamatergic neurons could alter anxiety‐like behaviors in anxious TBI, non‐anxious TBI, and Sham mice (Figure 4N–Q). These findings indicate that lOFC glutamatergic neurons play a pivotal role in modulating anxiety‐like behaviors in TBI mice.

### 2.5 Functional Connectivity and Causal Role of the aIC^Glu^‐lOFC^Glu^ Circuit in Anxiety‐Like Behaviors in TBI Mice

**FIGURE 5:**
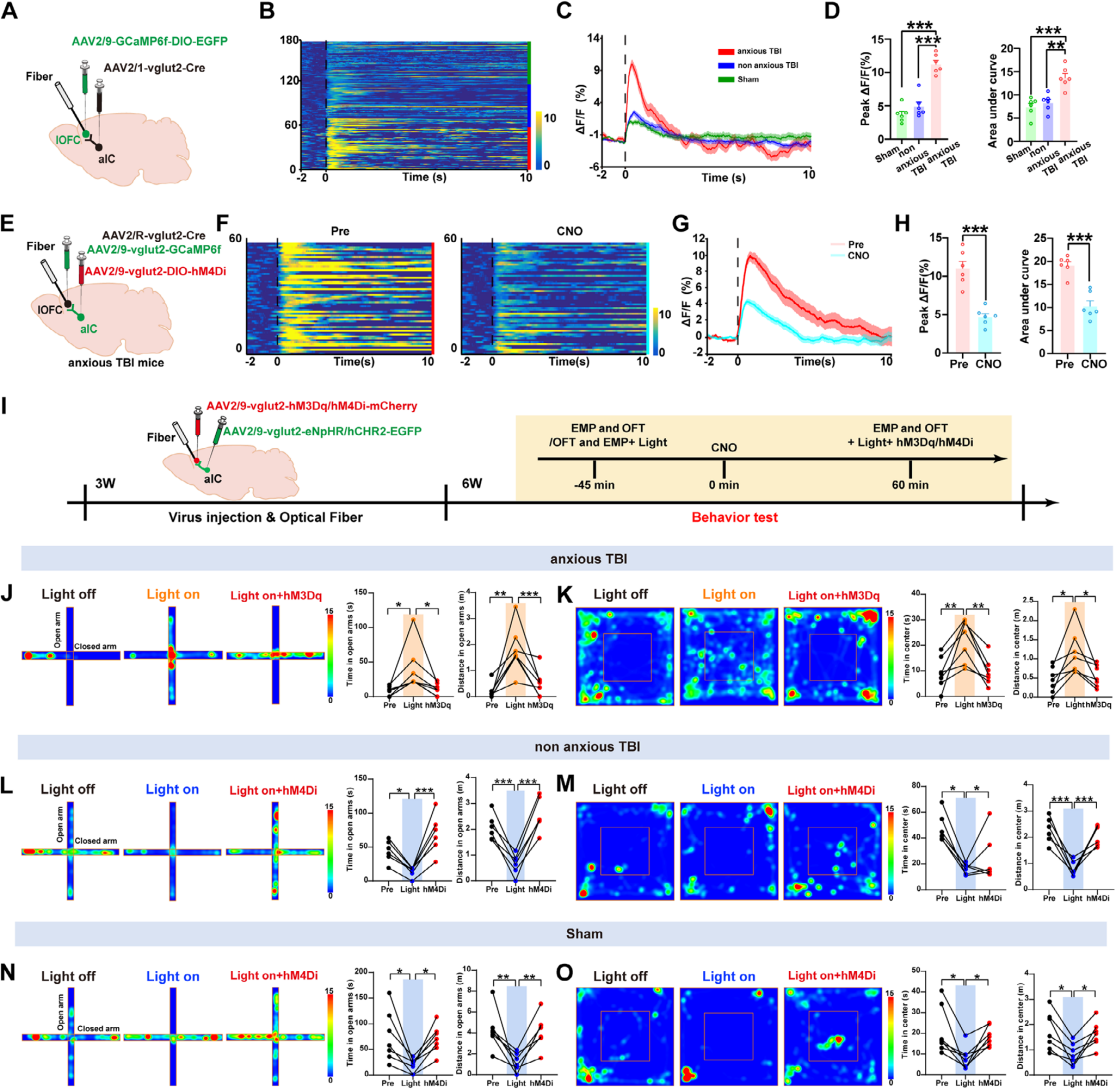
Critical role of the aIC‐lOFC glutamatergic circuit in modulating anxiety‐like behaviors in TBI mice. (A) Schematic diagram of viral injection sites. (B, C) Heatmaps (B) and representative calcium signal traces (C) of lOFC glutamatergic neurons receiving projections from aIC glutamatergic neurons in Sham, non‑anxious TBI, and anxious TBI mice. D) Statistical analysis of peak calcium signal activity and AUC in aIC glutamatergic neurons. (E) Diagram of viral injection sites. (F, G) Heatmaps (F) and averaged calcium traces (G) of lOFC glutamatergic neurons before (Pre) and after CNO administration. (H) Statistical analysis of peak and AUC of calcium signals in lOFC glutamatergic neurons under Pre and CNO conditions. (I) Schematic timeline of the combined optogenetic and chemogenetic manipulation and behavioral testing protocol. (J–O) Representative heatmaps and bar graphs of the EPM and OFT behavior test under different manipulation condition for the anxious TBI group (J, K), non‐anxious TBI (L, M) and Sham (N, O). Data are presented as mean ± SEM. Statistical analysis was performed using one‐way ANOVA followed by Tukey's multiple comparisons test, n = 6 per group, D; two‐tailed unpaired *t*‐test, n = 6 per group, H; two‐way ANOVA followed by Sidak's multiple comparisons test, n = 6–7 per group, (J—O); ns = not significant, *p < 0.05, **p < 0.01, ***p < 0.001.

In our previous experiments, we identified that glutamatergic neurons in the aIC and lOFC regulate anxiety‐like behaviors in anxious TBI mice. We further confirmed that glutamatergic neuronal terminals originating from the aIC directly project to glutamatergic neurons in the lOFC. To further validate the role of the aIC‐lOFC glutamatergic circuit in post‐TBI anxiety‐like behaviors, we injected AAV2/1‐vglut2‐Cre into the aIC and AAV2/9‐GCaMP6f‐DIO‐EGFP into the lOFC, followed by optical fiber implantation in the lOFC (Figure 5A). The results revealed that anxious TBI mice exhibited significantly enhanced calcium transient frequency and amplitude in glutamatergic neurons along the aIC‐lOFC pathway (Figure 5B–D). To determine whether the aIC‐lOFC glutamatergic circuit mediates anxiety‐like behaviors, we employed a combination of viral strategies and in vivo fiber photometry (Figure 5E). Calcium signals in the lOFC were recorded before (Pre) and after clozapine‑N‑oxide (CNO) administration. CNO‑induced chemogenetic inhibition of the aIC–lOFC circuit markedly reduced both the peak amplitude and the area under the curve (AUC) of calcium signals in lOFC glutamatergic neurons (Figure 5F–H), indicating an essential contribution of the aIC^Glu^‐lOFC^Glu^ circuit to anxiety‐related activity.

To directly test the causal role of this pathway in post‑TBI anxiety‐like behaviors, we employed a dual‑virus strategy combining optogenetic and chemogenetic manipulations to bidirectionally regulate glutamatergic activity within the aIC–lOFC pathway (Figure 5I). In anxious TBI mice, we injected the optogenetic virus AAV2/9‑vglut2‑eNpHR‑EGFP into the aIC and the chemogenetic virus AAV2/9‑vglut2‑hM3Dq‑mCherry into the lOFC, followed by optical fiber implantation in the lOFC. Optogenetic inhibition of aIC glutamatergic projections to the lOFC significantly alleviated anxiety‐like behaviors in the elevated plus maze and open field tests, as reflected by increased exploration of the open arms and the center zone, respectively. Importantly, this anxiolytic effect was abolished by CNO‑mediated activation of lOFC glutamatergic neurons via hM3Dq, restoring the anxiety‐like phenotype in anxious TBI mice (Figure 5J,K).

We further examined the behavioral consequences of activating the aIC–lOFC glutamatergic circuit. In Sham and non‑anxious TBI mice, we injected AAV2/9‑vglut2‑hChR2‑EGFP into the aIC and AAV2/9‑vglut2‑hM4Di‑mCherry into the lOFC, with optical fibers implanted in the lOFC (Figure 5I). Optogenetic activation of aIC glutamatergic projections to the lOFC induced robust anxiety‐like behaviors in both Sham and non‑anxious TBI mice, as evidenced by reduced time spent and distance traveled in the open arms of the EPM and in the center area of the OFT. This effect was reversed by CNO‑induced chemogenetic inhibition of lOFC glutamatergic neurons via hM4Di, leading to a marked attenuation of anxiety‐like behaviors in these mice (Figure 5L–O). To directly assess glutamate dynamics in the lOFC, we further injected AAV‑hSyn‑iGluSnFR‑WPRE‑pA into the lOFC (Figure S4A). We observed significantly elevated glutamate neurotransmitter levels in the lOFC of anxious TBI mice compared to non‐anxious TBI and Sham mice (Figure S4B–D). Together, these findings establish a direct aIC‐lOFC glutamatergic circuit whose activity drives anxiety‐like behaviors in TBI mice.

### 2.6 Downregulation of KCNC3 in Glutamatergic Neurons of the aIC in Anxious TBI Mice

**FIGURE 6:**
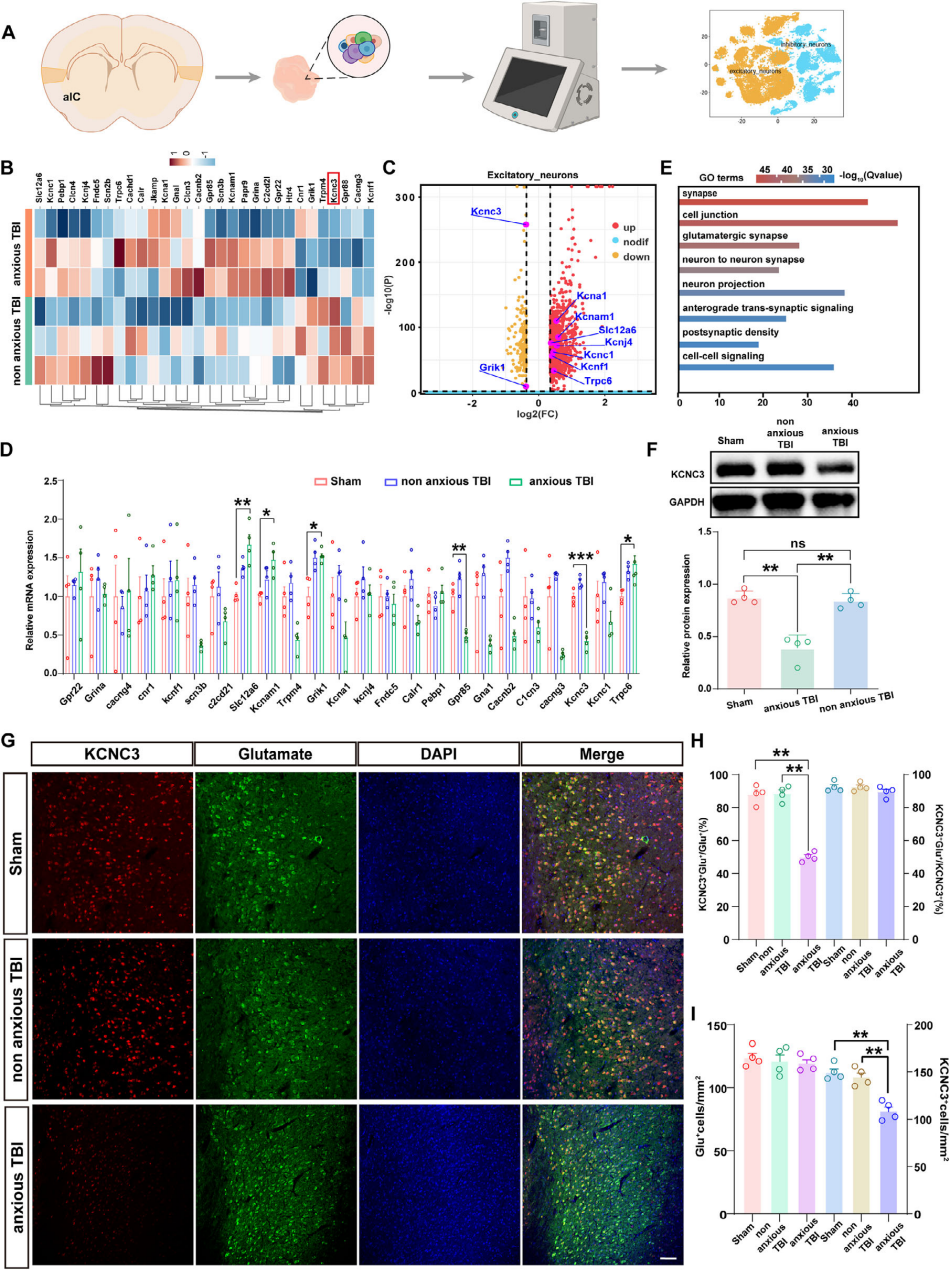
Downregulation of KCNC3 in aIC glutamatergic neurons of anxious TBI mice. (A) Schematic of the single‐cell sequencing workflow for the aIC. (B) Heatmap showing the distribution of significantly differentially expressed genes in aIC glutamatergic neurons. (C) Volcano plot illustrating gene distribution in aIC glutamatergic neurons. (D) RT‐qPCR statistical analysis of significantly differentially expressed genes. (E) GO analysis of the KCNC3 molecule. (F) Immunoblot and quantitative analysis of KCNC3 protein expression in the aIC. (G–I) Representative image showing co‐localization of KCNC3 with glutamatergic neurons in the aIC (G), scale bar, 50 µm), statistical analysis of the proportion of KCNC3 co‐labeling with glutamatergic neurons (H) or bar graph depicting expression levels of Glutamate and KCNC3 in the aIC (I). Data are presented as mean ± SEM. Statistical analysis was performed using one‐way ANOVA followed by Tukey's multiple comparisons test, n = 4 per group, D, F, H, I; ns = not significant, ^*^ *p* < 0.05, ^**^ *p* < 0.01, ^***^ *p* < 0.001.

To elucidate the molecular mechanisms by which the aIC‐lOFC circuit regulates post‐traumatic anxiety, we conducted single‐cell transcriptomic analysis of the aIC in both anxious and non‐anxious TBI mice (Figure 6A). Cell clustering and annotation based on t‐SNE projection and canonical marker gene expression delineated major neuronal and glial populations within the aIC (Figure S5A,B). Comparative analysis revealed a distinct ion channel‐related genes expression profile in glutamatergic neurons of the anxious TBI group compared to the non‐anxious TBI group, with prominent changes in KCNC3, KCNC1, and TRPC6 (Figure 6B,C). Consistent with these data, RT‐qPCR analysis of the aIC confirmed a significant downregulation of KCNC3 expression in anxious TBI mice, alongside upregulation of KCNMA1, TRPC6, and SLC12a6 (Figure 6D). Meanwhile KCNC3 expression showed the most pronounced alteration. Differential expression analysis further revealed that KCNC3 was selectively and robustly dysregulated across distinct cell populations, including inhibitory neurons, astrocytes, microglia, and OPCs (Figure S5C), suggesting a cell type‐specific contribution of KCNC3 to aIC circuit function. KCNC3 may collectively modulate the excitability of glutamatergic neurons in the aIC (Figure 6E). Thereby affecting the function of the aIC‐lOFC glutamatergic circuit and inducing post‐traumatic anxiety.

Western blot analysis further confirmed a significant reduction in KCNC3 protein levels in the aIC of anxious TBI mice compared to Sham and non‐anxious TBI (Figure 6F). Immunofluorescence co‐localization demonstrated that KCNC3 is predominantly expressed in glutamatergic neurons within the aIC (Figure 6G), and its expression was markedly reduced in the aIC glutamatergic neurons of anxious TBI mice (Figure 6H,I). Together, these findings suggest that downregulation of the potassium channel KCNC3 may lead to reduced membrane hyperpolarization in aIC glutamatergic neurons, enhancing neuronal excitability and glutamate release. This increased excitability is likely to potentiate the functional connectivity of the aIC–lOFC glutamatergic circuit and ultimately contribute to the manifestation of anxiety‑like behaviors following TBI.

### 2.7 Manipulating KCNC3 Expression in aIC Glutamatergic Neurons Altered Anxiety‐Like Behaviors

**FIGURE 7:**
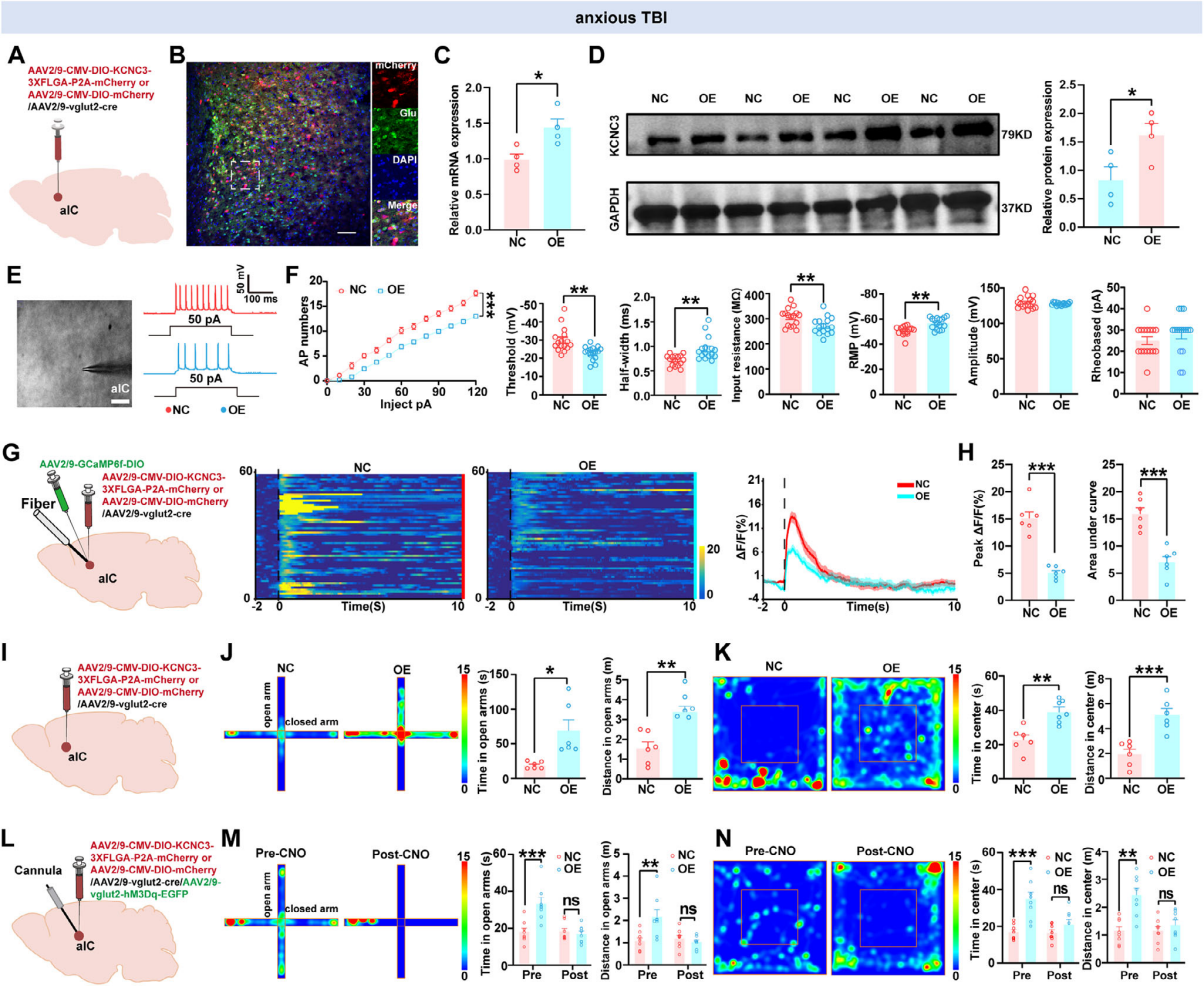
Overexpression of KCNC3 decreases the excitability of aIC glutamatergic neurons and attenuates anxiety‐like behavior in anxious TBI mice (A, B) Representative fluorescence images of KCNC3‐ overexpression (OE) or negative control (NC) glutamatergic neurons in the aIC of anxious TBI mice (scale bar: 50 µm). (C, D) Bar graphs showing relative KCNC3 mRNA (C) and protein (D) expression levels in the aIC of anxious TBI mice injected with KCNC3 overexpression or control virus. (E) Schematic of whole‐cell recording from aIC glutamatergic neurons and representative traces of action potentials (APs) elicited by 50 pA current injections in NC (anxious TBI mice injected with control virus) and OE (anxious TBI mice injected with KCNC3 overexpression virus) groups. (F) Summary of intrinsic membrane and firing properties of glutamatergic neurons, including AP numbers, AP threshold, AP half‐width, input resistance, resting membrane potential (RMP), AP amplitude and rheobase, AP amplitude, and rheobase in glutamatergic neurons. (G) Schematic of viral injection and optical fiber implantation in the aIC, and representative heatmaps and traces of calcium activity in glutamatergic neurons during open arm exploration in the EPM in NC and OE mice. (H) Quantification of peak calcium signals and AUC during open arm exploration. (I–K) Experimental design for behavioral testing (I) and representative EPM heatmaps with corresponding bar graphs of time spent and distance traveled in the open arms (J), as well as OFT trajectories with bar graphs of time spent and distance traveled in the center (K), in anxious TBI mice injected with KCNC3 overexpression or control virus. (L–N) Schematic of viral injection sites (L), representative EPM trajectory heatmaps before (Pre‐CNO) and after (Post‐CNO) CNO administration in anxious TBI mice, with corresponding bar graphs of time spent and distance traveled in the open arms (M), and heatmaps of OFT trajectories before and after CNO with bar graphs of time spent and distance traveled in the central area (N). Data are presented as mean ± SEM. Statistical analysis was performed using two‐tailed unpaired *t*‐test, n = 4, 6 or 7 per group, C, B, D, F, H, J, K; Wilcoxon signed‐rank test and two‐tailed unpaired *t*‐test, n = 6 per group, J; two‐way ANOVA followed by Sidak's multiple comparisons test, n = 6–7 per group, F (AP numbers), M, N; ns = not significant, ^*^ *p* < 0.05, ^**^ *p* < 0.01, ^***^ *p* < 0.001.

KCNC3, a key subunit of voltage‐gated potassium channels, has been reported to enhance excitatory neurotransmitter release and potentiate glutamatergic synaptic transmission [27]. To investigate the functional role of KCNC3 in anxiety‐like behaviors following TBI, we selectively overexpressed KCNC3 in glutamatergic neurons of the aIC in anxious TBI mice using viral‐mediated gene delivery (Figure 7A,B). Consistently, RT‐qPCR and Western blot analyses revealed significantly elevated KCNC3 mRNA and protein levels in the aIC (Figure 7C,D). We next examined whether KCNC3 overexpression alters the intrinsic excitability of aIC glutamatergic neurons. Whole‐cell current‐clamp recordings were performed from glutamatergic neurons in acute aIC slices of anxious TBI mice injected with control or KCNC3‐overexpression virus (Figure 7E). Compared with the NC group, neurons in the KCNC3 OE group displayed a significant reduction in action potential numbers and input resistance, accompanied by an increase in action potential threshold, action potential half‐width, and resting membrane potential (RMP), indicating that glutamatergic neurons in the KCNC3 OE group exhibit reduced intrinsic excitability (Figure 7F).

To determine how KCNC3 overexpression affects neuronal activity in vivo during anxiety‐related behavior, we performed fiber photometry recordings of calcium dynamics in aIC glutamatergic neurons during the EPM testing, as reflected by decreased peak and AUC (Figure 7G,H). We then assessed whether KCNC3 overexpression modulates anxiety‐like behavior (Figure 7I). Behavioral assessments revealed that anxious TBI mice overexpressing KCNC3 spent significantly more time and traveled greater distances on the open arms of the EPM, as well as in the center area of the OFT, supporting an anxiolytic effect of KCNC3 overexpression in anxious TBI mice (Figure 7J,K). To further investigate the relationship between KCNC3 and glutamatergic neuronal activity, we co‐injected the KCNC3 overexpression virus targeting glutamatergic neurons with a chemogenetic activator virus into the aIC of anxious TBI mice (Figure 7L). Before CNO administration, these mice showed significantly increased time spent and distance travel in the EPM open arms and OFT center compared to the control virus group, consistent with reduced anxiety‐like behaviors. However, following CNO administration, these behaviors were reversed, with time and distance in the EPM open arms and OFT center were significantly reduced, approaching the levels observed in NC mice, indicating a restoration of anxiety‐like behaviors (Figure 7M,N).

### 2.8 Knockdown of KCNC3 in aIC Glutamatergic Neurons Increases Neuronal Excitability and Induces Anxiety‐Like Behaviors in Non‐Anxious TBI Mice

**FIGURE 8:**
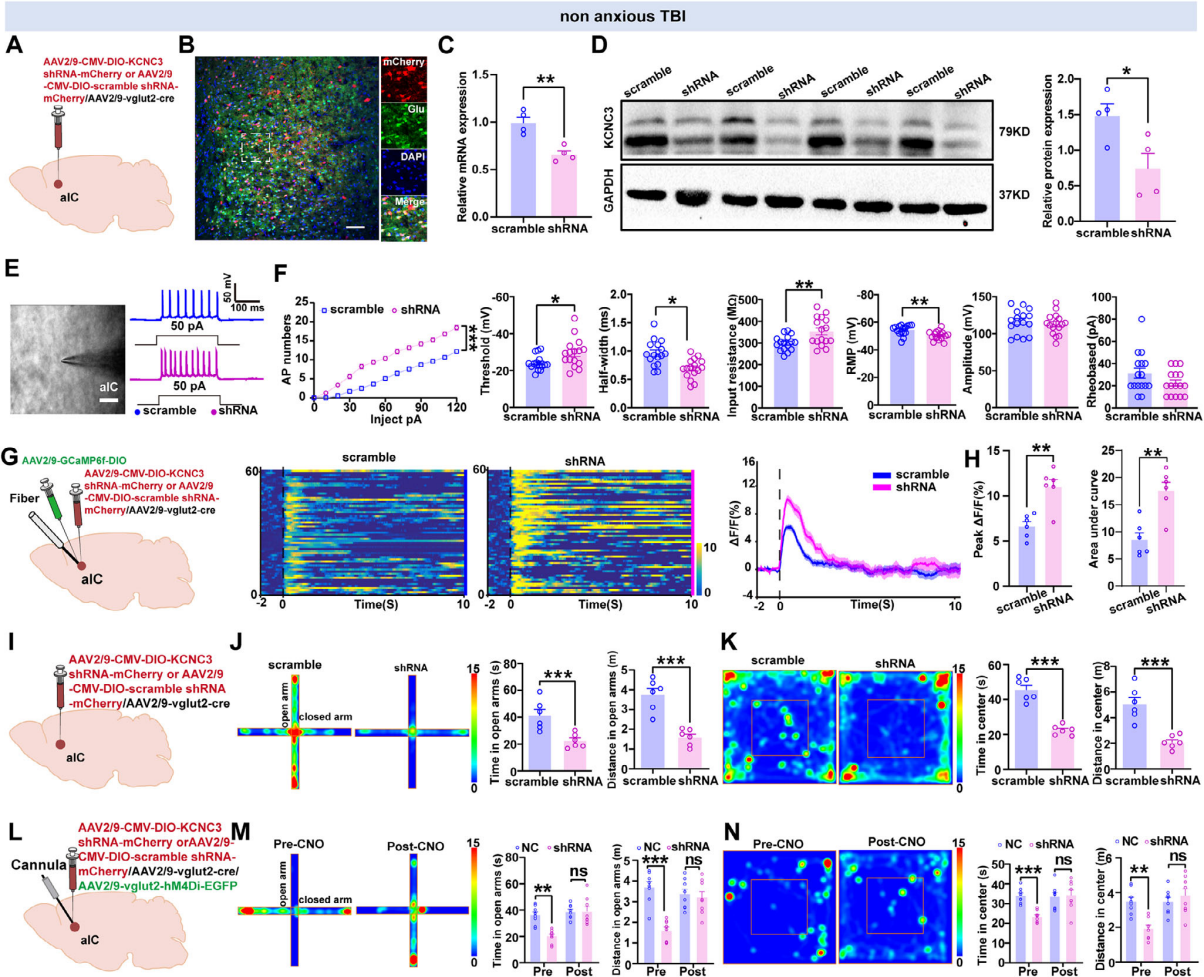
Knockdown of KCNC3 enhances the excitability of aIC glutamatergic neurons and increases anxiety‐like behavior in non‐anxious TBI mice. (A, B) Fluorescence micrographs of KCNC3 knockdown (shRNA) or scramble control groups in glutamatergic neurons within the aIC of non‐anxious TBI mice (scale bar, 50 µm). (C, D) Bar graph depicting KCNC3 mRNA and protein expression levels in the aIC following KCNC3 knockdown and control virus injection. (E) Schematic illustration of electrode placement and representative traces of action potentials (Aps) elicited by 50 pA current injections of glutamatergic neurons in the scramble mice (non‐anxious TBI mice injected with control virus) and shRNA mice (non‐anxious TBI mice injected with knockdown virus). (F) Summary of intrinsic membrane and firing properties of glutamatergic neurons, including AP numbers, AP threshold, AP half‐width, input resistance, RMP, AP amplitude, and rheobase in glutamatergic neurons. (G) Diagram of viral injection sites within the aIC, along with a heatmap and line graph of calcium activity in glutamatergic neurons during exploration of the open arms of the EPM in non‐anxious TBI mice after KCNC3 knockdown and control virus injection. (H) Statistical analysis of peak calcium signals and AUC during EPM open arm exploration. (I) Diagram of viral injection sites. (J, K) Heatmap representations and bar graphs showing the time spent and distance traveled in the open arms during EPM testing (J), as well as OFT trajectories and time spent and distance traveled in the center (K), in non‐anxious TBI mice injected with KCNC3 knockdown (shRNA) and control virus (scramble). (L–N) Diagram of viral injection sites (L), heatmap of EPM trajectories before (Pre‐CNO) and after (Post‐CNO) CNO administration in non‐anxious TBI mice, with corresponding bar graphs of time spent and distance traveled in the open arms (M), and heatmaps of OFT trajectories before and after CNO with bar graphs of time spent and distance traveled in the central area (N). Data are presented as mean ± SEM. Statistical analysis was performed using two‐tailed unpaired *t*‐test, n = 4, 6 or 7 per group, C, D, F, J, K; Wilcoxon signed‐rank test and two‐tailed unpaired *t*‐test, n = 6 per group, H; two‐way ANOVA followed by Sidak's multiple comparisons test, n = 6–7 per group, F (AP numbers), M, N; ns = not significant, ^*^ *p* < 0.05, ^**^ *p* < 0.01, ^***^ *p* < 0.001.

Given that KCNC3 overexpression in aIC glutamatergic neurons alleviated anxiety‐like behaviors in anxious TBI mice, we next examined whether reducing KCNC3 expression is sufficient to induce anxiety‐like phenotypes in non‐anxious TBI mice. To this end, we knocked down KCNC3 specifically in aIC glutamatergic neurons using an shRNA‐based viral approach (Figure 8A,B). Consistently, KCNC3 mRNA and protein levels were significantly decreased in the knockdown group (Figure 8C,D). We then investigated whether KCNC3 knockdown altered the intrinsic properties of aIC glutamatergic neurons. Whole‐cell current‐clamp recordings were performed in acute aIC slices from non‐anxious TBI mice injected with scramble or KCNC3 shRNA virus (Figure 8E). Compared with the scramble group, neurons in the KCNC3 KD group showed an increase in action potential firing frequency and input resistance, together with a significant decrease in action potential threshold, action potential half‐width, and resting RMP, indicating that KCNC3 knockdown enhances the intrinsic excitability of aIC glutamatergic neurons in non‐anxious TBI mice (Figure 8F).

To determine how KCNC3 knockdown impacts neuronal activity in vivo during anxiety‐related behavior, we performed fiber photometry recordings of calcium dynamics in aIC glutamatergic neurons during exploration of the open arms in the EPM (Figure 8G). Statistical analysis confirmed significantly higher peak calcium signals and greater AUC in the KCNC3 knockdown group, indicating enhanced aIC activity during anxiety‐related exploration (Figure 8H). We next evaluated whether KCNC3 knockdown in aIC glutamatergic neurons is sufficient to induce anxiety‐like behavior at the behavioral level (Figure 8I). Behavioral assessments revealed that KCNC3 knockdown mice spent less time in, and traveled shorter distances within, the open arms compared with scramble controls (Figure 8J). Similarly, KCNC3 knockdown mice exhibited significantly reduced time spent and distance traveled in the center area of the OFT relative to controls (Figure 8K). These findings indicate that KCNC3 knockdown in aIC glutamatergic neurons converts non‐anxious TBI mice into an anxiety‐like behavioral state.

To further validate the relationship between KCNC3 and neuronal excitability, we co‐injected the KCNC3 knockdown virus targeting glutamatergic neurons with a chemogenetic inhibitory virus into the aIC of non‐anxious TBI mice (Figure 8L). Prior to CNO administration, these mice showed significantly reduced time and distance in the OFT center and EPM open arms compared to the control virus group, suggesting a significant increase in anxiety‐like behaviors. Following CNO administration, these behaviors were reversed, with significantly increased time spent and distance traveled in the OFT center and EPM open arms compared with Pre‐CNO levels (Figure 8M,N).

### 2.9 Knockdown of KCNC3 in aIC Glutamatergic Neurons Increases Neuronal Excitability and Induces Anxiety‐Like Behaviors in Sham Mice

**FIGURE 9:**
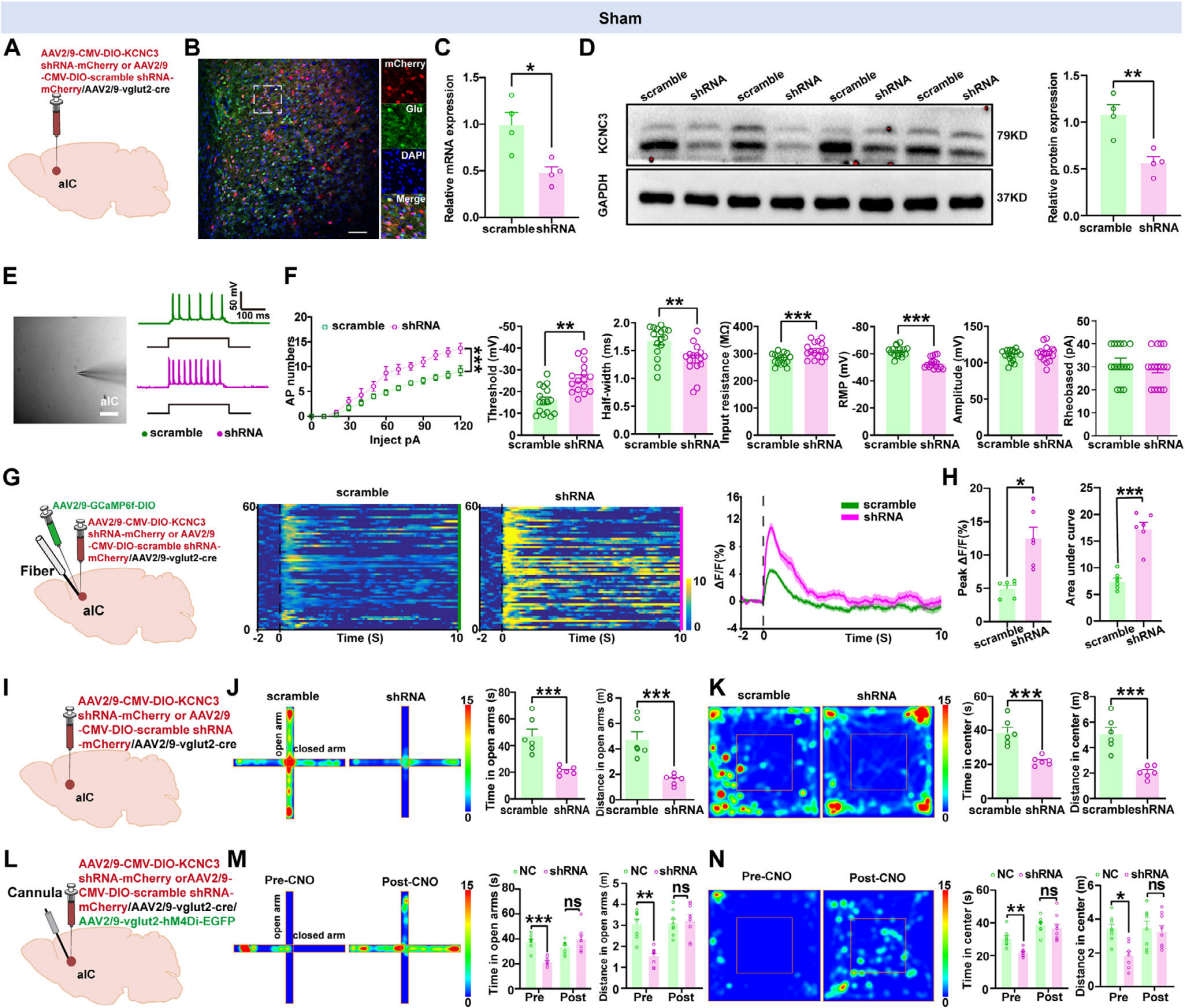
Knockdown of KCNC3 enhances the excitability of aIC glutamatergic neurons and increases anxiety‐like behavior in Sham mice. (A, B) Fluorescence micrographs of KCNC3 knockdown or scramble control groups in glutamatergic neurons within the aIC of Sham mice (scale bar, 50 µm). (C, D) Bar graph depicting mRNA and protein expression levels in the aIC following KCNC3 knockdown and control virus injection. (E) Schematic illustration of electrode placement and representative traces of AP elicited by 50 pA current injections of glutamatergic neurons in the scramble mice (non‐anxious TBI mice injected with control virus) and shRNA mice (Sham mice injected with knockdown virus). (F) Summary of intrinsic membrane and firing properties of glutamatergic neurons, including AP numbers, AP threshold, AP half‐width, input resistance, RMP, AP amplitude and rheobase in glutamatergic neurons. (G) Diagram of viral injection sites within the aIC, along with a heatmap and line graph of calcium activity in glutamatergic neurons during exploration of the open arms of the EPM in Sham mice after KCNC3 knockdown and control virus injection. (H) Statistical analysis of peak calcium signals and AUC during EPM open arm exploration. (I) Diagram of viral injection sites. (J, K) Heatmap representations and bar graphs showing the time spent and distance traveled in the open arms during EPM testing (J), as well as OFT trajectories and time spent and distance traveled in the center (K), in non‐anxious TBI mice injected with KCNC3 knockdown (shRNA) and control virus (scramble). (L–N) Diagram of viral injection sites (L), heatmap of EPM trajectories before (Pre‐CNO) and after (Post‐CNO) CNO administration in non‐anxious TBI mice, with corresponding bar graphs of time spent and distance traveled in the open arms (M), and heatmaps of OFT trajectories before and after CNO with bar graphs of time spent and distance traveled in the central area (N). Data are presented as mean ± SEM. Statistical analysis was performed using two‐tailed unpaired *t*‐test, n = 4, 6 or 16 per group, B, D, F, J, K; Wilcoxon signed‐rank test and two‐tailed unpaired *t*‐test, n = 6 per group, H; two‐way ANOVA followed by Sidak's multiple comparisons test, n = 6–7 per group, F (AP numbers), M, N; ns = not significant, ^*^ *p* < 0.05, ^**^ *p* < 0.01, ^***^ *p* < 0.001.

To determine whether the effects of KCNC3 knockdown on aIC glutamatergic neurons and anxiety‐like behavior are specific to the TBI condition or reflect a more general mechanism, we performed parallel manipulations in Sham mice. KCNC3 expression was selectively reduced in aIC glutamatergic neurons of Sham mice using a viral shRNA strategy (Figure 9A,B). Consistently, KCNC3 mRNA and protein levels were significantly decreased (Figure 9C,D). We next examined whether KCNC3 knockdown in Sham mice produced similar changes in intrinsic neuronal properties as observed in non‐anxious TBI mice. Whole‐cell current‐clamp recordings were conducted in acute aIC slices from Sham mice injected with control or KCNC3 shRNA virus (Figure 9E). Compared with the scramble group, neurons in the KCNC3 KD group exhibited a significant increase in action potential firing frequency and input resistance, together with a significant decrease in action potential threshold, action potential half‐width, and resting RMP, indicating higher intrinsic excitability in the KCNC3 KD group (Figure 9F).

To assess the impact of KCNC3 knockdown on in vivo neuronal activity during anxiety‐related behavior, we performed fiber photometry recordings of calcium dynamics in aIC glutamatergic neurons during EPM exploration in Sham mice (Figure 9G). Statistical analysis confirmed significantly higher peak calcium signals and a greater AUC in the KCNC3 KD group, indicating enhanced aIC activity during anxiety‐related exploration (Figure 9H). We then evaluated whether KCNC3 knockdown in aIC glutamatergic neurons of Sham mice is sufficient to induce anxiety‐like behavior (Figure 9I). Behavior assessments disclosed the knockdown group exhibited significantly reduced time and distance spent in the OFT center and the EPM open arms, indicating a significant increase in anxiety‐like behaviors (Figure 9J,K).

To further validate the link between KCNC3‐dependent regulation of glutamatergic neuronal activity and anxiety‐like behavior, we co‐injected KCNC3 knockdown virus together with a chemogenetic inhibitory virus into the aIC of Sham mice (Figure 9L). Prior to CNO administration, these mice showed significantly reduced time and distance in the OFT center and EPM open arms compared with the control virus group, consistent with increased anxiety‐like behavior. Following CNO administration, these behaviors were reversed, with significantly increased time and distance in the OFT center and EPM open arms relative to their Pre‐CNO levels (Figure 9M,N).

Collectively, these findings identify KCNC3 as a critical regulator of aIC glutamatergic neuronal excitability. Functionally, KCNC3 downregulation enhances neuronal activity and drives anxiety‐like behaviors, whereas KCNC3 overexpression dampens neuronal activity and ameliorates anxiety‐related phenotypes. These results position KCNC3 as a pivotal molecular node in the pathophysiology of post‐traumatic anxiety and highlight its potential as a promising therapeutic target. Moreover, they are in line with accumulating evidence implicating voltage‐gated potassium channels, particularly the Kv3 subfamily, in a broad spectrum of neurological and psychiatric disorders [28, 35].

### 2.10 Glutamatergic aIC ^KCNC3+^‐lOFC Circuit Regulates Anxiety‐Like Behaviors

**FIGURE 10:**
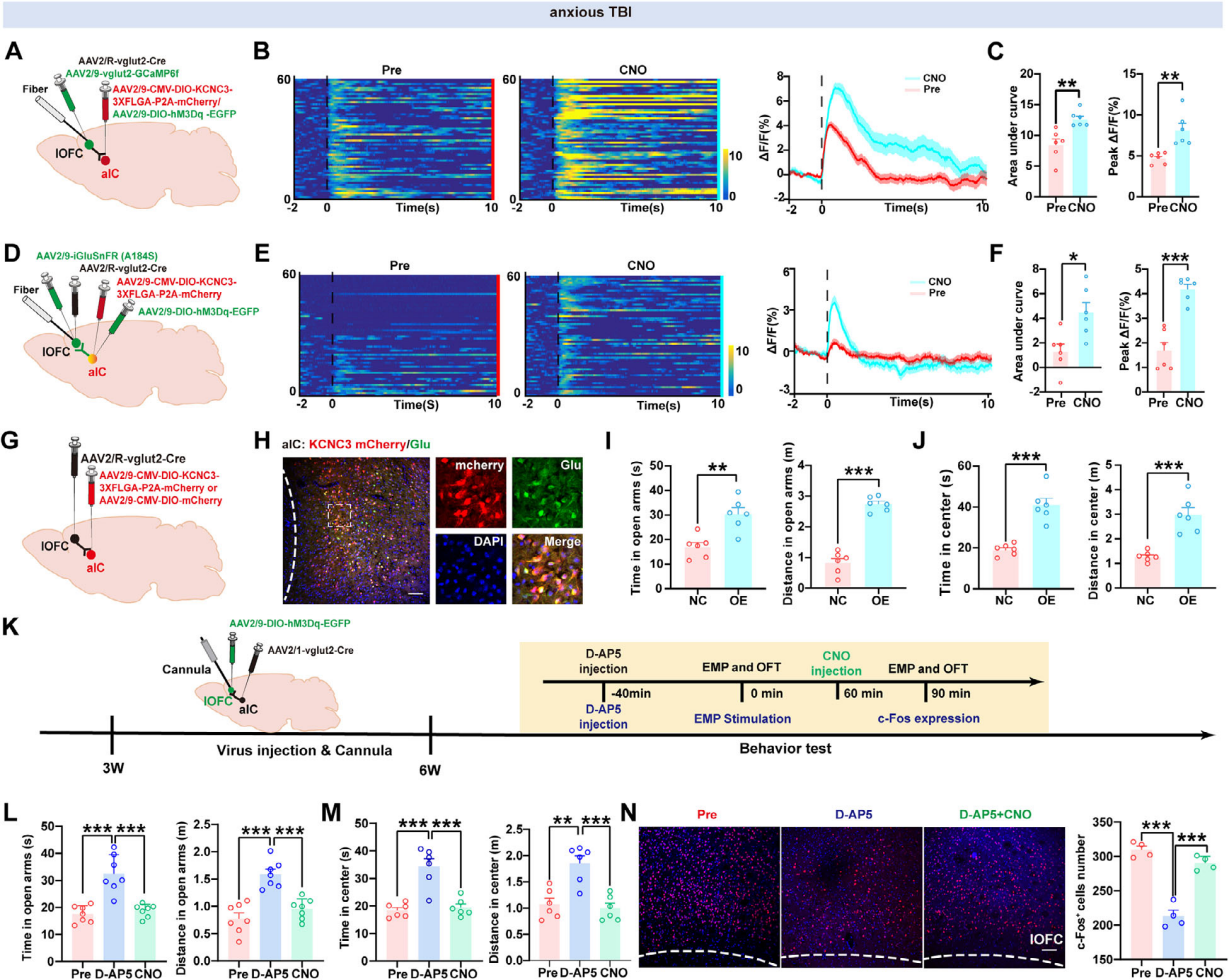
Regulation of anxiety‐like behaviors by the aICKCNC3+‐lOFC glutamatergic circuit in anxious TBI mice. (A–F) Viral strategy for detecting Ca^2+^ (A) and glutamate (D) levels in the lOFC with aIC^KCNC3+^‐lOFC circuit activation in anxious mice, representative heatmap, curve graph of lOFC Ca^2+^ (B) and glutamate (E) levels before and after aIC^KCNC3+^‐lOFC circuit inhibition, and bar graph of peak calcium signals and AUC of lOFC Ca^2+^ (C) glutamate (F) levels before and after aIC^KCNC3+^‐lOFC circuit inhibition. (G–J) Schematic of viral injections in anxious TBI mice (G); representative image showing KCNC3 overexpression and co‐localization with glutamatergic neurons in the aIC (H, scale bar, 50 µm), and bar graph illustrating EPM (I) and OFT (J) behavioral outcomes following KCNC3 overexpression in the aIC. (K–M) Schematic of the experimental procedure (K) and bar graph of illustrating EPM (L) and OFT (M) behavioral outcomes under Pre, D‐AP5, and Post‐CNO in anxious TBI mice. (N) Representative immunofluorescence images and quantitative statistical analysis of c‐Fos‐positive cell expression in the aIC under Pre, D‐AP5, and D‐AP5+CNO conditions following EPM exposure (scale bar, 100 µm). Data are presented as mean ± SEM. Statistical analysis was performed using a two‐tailed unpaired *t*‐test, n = 6 per group, C, F, I, J; one‐way ANOVA followed by Tukey's multiple comparisons test, n = 6 per group, L, M, N; ns = not significant, ^*^ *p* < 0.05, ^**^ *p* < 0.01, ^***^ *p* < 0.001.

To investigate whether the aIC^KCNC3+^‐lOFC glutamatergic circuit mediates anxiety‐like behaviors, we employed a combination of viral strategies and in vivo fiber photometry (Figure 10A). Calcium signals of aIC^Glu^ were recorded under Pre and CNO administration (Figure 10B,C). Activation of the aIC^KCNC3+^‐lOFC circuit significantly enhanced calcium signals of lOFC^Glu^ (Figure 10C). To assess glutamate release in response to EPM stimulation in anxious TBI mice, AAV2/9‐iGluSnFR (A184S) and AAV2/R‐vglut2‐Cre were injected in lOFC, while AAV2/9‐CMV‐DIO‐KCNC3‐3XFLGA‐P2A‐mCherry and AAV2/9‐DIO‐hM3Dq‐EGFP were injected in aIC (Figure 10D). Activation of aIC^KCNC3+^‐lOFC glutamatergic circuit with CNO increased glutamate release in the lOFC in the presence of overexpression of KCNC3 in aIC (Figure 10E,F). To further examine the contribution of this pathway in non‐anxious conditions, AAV2/9‐iGluSnFR (A184S) and AAV2/R‐vglut2‐Cre were injected in lOFC, AAV2/9‐CMV‐DIO‐KCNC3‐3XFLGA‐P2A‐mCherry together with AAV2/9‐DIO‐hM4Di‐EGFP were injected in aIC in non‐anxious TBI and Sham mice. Inhibition of aIC^KCNC3+^‐lOFC glutamatergic circuit reduced glutamate release in the lOFC while knockdown of KCNC3 in the aIC (Figures S6A–C and S7A–C), suggesting that EPM stimulation promotes glutamate release from aIC^KCNC3+^‐lOFC glutamatergic circuits.

To further validate whether KCNC3 modulates anxiety‐like behaviors in anxious TBI mice via the aIC‐lOFC glutamatergic circuit, we specifically overexpressed KCNC3 in aIC neurons projecting to lOFC glutamatergic neurons. AAV2/9‐CMV‐DIO‐KCNC3‐3XFLAG‐P2A‐mCherry or AAV2/9‐CMV‐DIO‐mCherry was injected into the aIC, and AAV2/R‐vglut2‐Cre into the lOFC (Figure 10G). Immunofluorescence showed co‐localization of KCNC3‐positive mCherry neurons with glutamate neurons in the aIC following KCNC3 overexpression (Figure 10H). Behavioral assays demonstrated that selective KCNC3 overexpression in aIC‐lOFC glutamatergic neurons significantly attenuated anxiety‐like behaviors in anxious TBI mice (Figure 10I,J). These data indicate that KCNC3 in aIC glutamatergic neurons regulates anxiety‐like behaviors in anxious TBI mice via projections to the lOFC. Similarly, to further confirm the role of KCNC3 in the aIC‐lOFC glutamatergic circuit in regulating anxiety‐like behaviors, we specifically knocked down KCNC3 in aIC glutamatergic neurons of Sham and non‐anxious TBI mice. We injected AAV2/9‐DIO‐KCNC3‐shRNA‐mCherry or AAV2/9‐DIO‐scramble‐shRNA‐mCherry into the aIC, and AAV2/R‐vglut2‐Cre into the lOFC. Compared to the control virus group, KCNC3 knockdown increased anxiety‐like behaviors in Sham and non‐anxious TBI mice (Figures S7D–G and S8D–G).

Collectively, these findings demonstrate that KCNC3 plays a critical role in modulating anxiety‐like behaviors through the aIC‐lOFC glutamatergic circuit. Overexpression of KCNC3 in aIC glutamatergic neurons projecting to the lOFC alleviates anxiety‐like behaviors, whereas its knockdown exacerbates these behaviors. This highlights the therapeutic potential of targeting the aIC^KCNC3+^‐lOFC glutamatergic circuit for anxiety‐like behaviors, particularly in the context of traumatic brain injury.

Furthermore, we examined whether glutamatergic signaling within the aIC–lOFC circuit further modulates anxiety‐like behaviors in anxious TBI mice. AAV2/1‐vglut2‐cre was injected into the aIC and AAV2/9‐DIO‐hM3Dq‐EGFP into the lOFC, with cannulas implanted in the lOFC (Figure 10K). Initially, we performed behavioral assessments on anxious TBI mice. Subsequently, we administered the selective NMDAR antagonist D‐AP5 into the lOFC and conducted anxiety‐like behavioral evaluations 1 h later. The results indicated a reduction in anxiety‐like behaviors in the anxious TBI mice. Following this, CNO was injected into the lOFC, and we re‐evaluated the anxiety‐like behaviors in anxious TBI mice after 1 h, revealing a restoration of anxiety‐like behaviors (Figure 10L,M).

Using the same viral strategy in anxious TBI mice, we administered D‐AP5 and a combination of D‐AP5 and CNO into the lOFC. The mice underwent EPM testing 1 h after the infusion of D‐AP5 or the combination of D‐AP5 and CNO, followed by a 2‐h perfusion with paraformaldehyde to assess c‐Fos‐positive cell expression in the lOFC. D‐AP5 injection significantly decreased the number of c‐Fos‐positive cells in the lOFC compared with untreated mice, whereas co‐administration of D‐AP5 and CNO significantly increased c‐Fos‐positive cell expression compared with D‐AP5 alone (Figure 10N). These findings highlight an intricate interplay between glutamatergic signaling and neuronal activity in the aIC‐lOFC circuit, shedding light on potential mechanisms underlying anxiety disorders associated with traumatic brain injury. Providing potential new avenues for therapeutic intervention in anxiety‐related disorders.

## 3 Discussion

**FIGURE 11:**
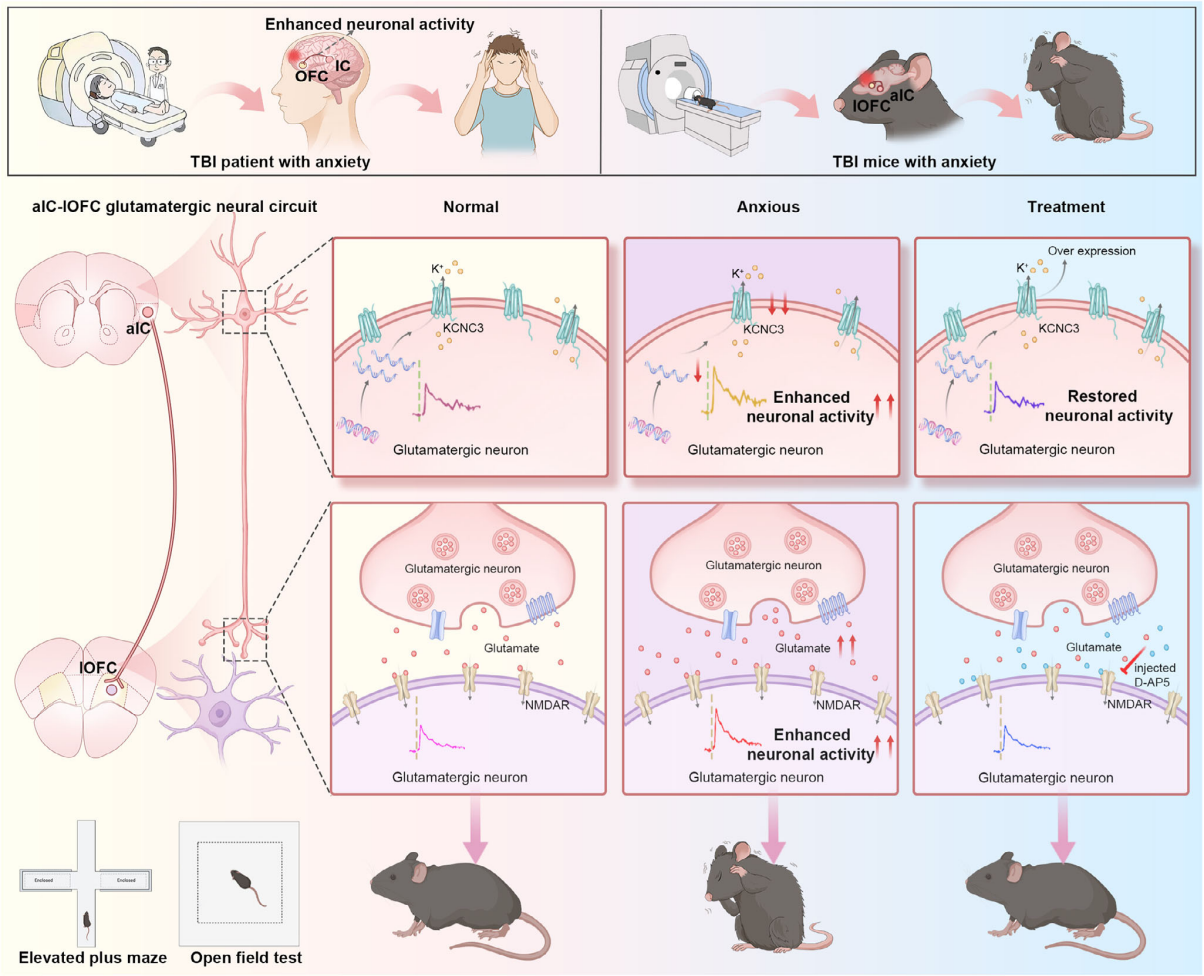
Schematic model of the mechanism by which the aIC‐lOFC glutamatergic circuit regulates post‐TBI anxiety.

TBI represents a significant global health challenge, with a substantial proportion of patients developing anxiety disorders that profoundly hinder functional recovery and diminish quality of life [36, 37]. In this study, we focused on patients with TBI‐induced anxiety following frontal lobe injury, revealing for the first time that both neuronal activity and functional connectivity between the aIC and lOFC are markedly enhanced. Importantly, our findings identified that the heightened activity of the aIC‐lOFC glutamatergic circuit plays a crucial role in the regulation of anxiety‐like behaviors in TBI mice with frontal lobe injury. Furthermore, we first identified that the specific downregulation of KCNC3 expression in aIC glutamatergic neurons leads to their activation, which subsequently induces hyperactivity of the aIC‐lOFC glutamatergic circuit, ultimately contributing to post‐traumatic anxiety (Figure 11). Collectively, our data establish KCNC3 as a druggable regulator of post‐TBI anxiety and provide a framework for developing circuit‐specific ion‐channel‐targeted therapeutics.

We first conducted a clinical study with well‐defined inclusion/exclusion criteria, enrolling 15 TBI patients with frontal lobe injuries and comorbid anxiety (HAMA score >14) and 15 age‐and gender‐matched non‐anxious TBI patients. Resting‐state BOLD‐fMRI analysis showed that anxious TBI patients exhibited significantly higher activity (assessed by ALFF) and functional connectivity in the aIC and lOFC compared to non‐anxious TBI patients (Figure 1 and Figure S1). This is consistent with previous neuroimaging studies showing structural and functional abnormalities in the insulo‐orbitofrontal network in anxiety disorders, but extends these findings by linking this network dysfunction specifically to TBI‐induced anxiety. This finding highlights the critical roles of the aIC and lOFC in emotion processing and regulation [38]. The aIC is considered a central region for integrating internal physiological states with external environmental information, while the lOFC plays a key role in decision‐making, reward processing, and emotional regulation [39, 40, 41]. Studies utilizing neuroimaging techniques have revealed structural and functional alterations within the aIC and lOFC in individuals experiencing anxiety symptoms and diagnosed anxiety disorders, including those comorbid with conditions like late‐life depression [20, 42, 43, 44].

In addition, the clinical cohort included only 15 TBI patients with anxiety and 15 matched non‐anxious TBI controls, which provides limited statistical power and increases the risk of both type I and type II errors, thereby constraining the robustness and generalizability of the observed aIC‐lOFC alterations. As a result, our human findings should be regarded as preliminary and primarily hypothesis‐generating, rather than definitive evidence for a clinically scalable biomarker. Future studies with larger, more heterogeneous and preferably multi‐centre cohorts, and with broader age ranges and sufficient sample sizes to model age‐by‐diagnosis and other interaction effects, will be essential to replicate these associations, to clarify potential moderator effects, and to determine the extent to which aIC‐lOFC circuit alterations can be generalized across different TBI populations and anxiety phenotypes. Despite these limitations, our data support the view that abnormal activity and connectivity between the aIC and lOFC may constitute a circuit basis for anxiety following TBI. The observed increased activity is also consistent with findings in other neurological conditions and TBI models where excitation/inhibition imbalance and network enhancement in neuronal activity are key features [45, 46, 47]. Furthermore, to minimize the impact of neurodevelopmental and aging‐related variability in brain structure and connectivity, we restricted enrollment to adults aged 18–50 years. This age range was also chosen because, in our clinical setting, the majority of TBI cases within this bracket arise from traumatic injuries sustained in construction‐site accidents, making this group the most representative of the patient population we routinely encounter. While this sampling strategy improves internal validity and enhances clinical relevance for this common TBI subgroup, it constrains generalizability of our findings to adolescent and older adult TBI populations. Future studies leveraging larger, more heterogeneous and ideally multi‐centre cohorts, with sufficient power to model age‐by‐diagnosis and other interaction effects, will be essential to replicate our observations and to determine the extent to which aIC‐lOFC circuit alterations generalize across TBI subtypes, age groups, and anxiety phenotypes.

To further validate the clinical findings and explore the underlying mechanisms, we established a mouse model of moderate frontal lobe injury. Behavioral assessments successfully distinguished mice exhibiting anxiety‐like behaviors from those not displaying such behavior. While we controlled for locomotor confounds, future studies using more sophisticated approaches (automated behavioral segmentation or operant tasks) could further dissociate anxiety‐like behavior from general activity. This study ultimately defined three groups: the Sham group, the non‐anxious TBI group, and the anxious TBI group. These experimental results robustly confirmed a clear causal relationship between frontal lobe injury and anxiety‐like behaviors, laying a solid foundation for subsequent in‐depth exploration of the molecular mechanisms and neural circuit. Consistent with clinical results, the anxious TBI mice exhibited enhanced neural activity in the aIC and lOFC, as confirmed by BOLD‐fMRI and fos protein expression analysis. More importantly, our findings further demonstrates the critical role of this specific aIC‐lOFC glutamatergic circuit in post‐TBI anxiety. By precisely modulating the aIC‐lOFC glutamatergic circuit using optogenetic and chemogenetic techniques, we successfully bidirectionally regulated anxiety‐like behaviors in mice, directly proving the causal relationship between circuit activity and anxiety levels. Although we detected only sparse co‐localization of c‐Fos with GABAergic neurons in the aIC and lOFC under our experimental conditions, this finding should not be taken to imply a negligible role for inhibitory circuits. Local GABAergic interneurons, as well as long‐range GABAergic inputs from other regions, are likely to contribute critically to the regulation of excitatory activity and to the maintenance of local excitatory–inhibitory (E/I) balance. Because the present study did not systematically characterize the inhibitory microcircuit architecture or specific interneuron subtypes, we cannot determine how these elements shape the observed pattern of predominantly glutamatergic activation in anxious TBI mice. Future work combining cell‐type–specific manipulation, in vivo recordings, and circuit tracing will be required to delineate how local and afferent GABAergic circuits interact with glutamatergic populations in the aIC and lOFC to influence anxiety‐related phenotypes after TBI.

To investigate the molecular mechanisms underlying the hyperactivity of the aIC‐lOFC glutamatergic circuit, we performed single‐cell sequencing on glutamatergic neurons in the aIC. Excitingly, our results revealed a specific downregulation of the *KCNC3* gene in the aIC glutamatergic neurons of anxious TBI mice. *KCNC3* gene encodes the Kv3.3 potassium channel subunit, which plays a crucial role in fast‐spiking neurons by regulating neuronal excitability and action potential firing patterns [28]. The downregulation of KCNC3 may alter the hyperpolarization‐activated gating properties of aIC glutamatergic neurons, thereby increasing their excitability. Our subsequent validation experiments supported this hypothesis: overexpression of KCNC3 in the aIC glutamatergic neurons of anxious TBI mice alleviated anxiety‐like behaviors, whereas konckdown in non‐anxious TBI and Sham mice increased anxiety‐like behaviors. These findings strongly suggest that the downregulation of KCNC3 is a key molecular event mediating the hyperexcitability of the aIC‐lOFC glutamatergic circuit, leading to post‐traumatic anxiety. By demonstrating how KCNC3 regulates post‐traumatic anxiety, we reveal potential therapeutic targets for addressing anxiety‐like behaviors in affected individuals, underscoring the importance of this potassium channel in emotional processing and neural health. Although we found that KCNC3 knockdown reduced action potential half‐width, which is inconsistent with previous studies, this discrepancy may be explained by earlier studies of Kv3.3 were conducted in Purkinje cells, auditory brainstem principal neurons/calyx terminals, and TRN fast‐spiking GABAergic neurons, in which Kv3.1/Kv3.3 provide the dominant repolarizing conductance required to sustain fast‐spiking firing patterns [27, 48, 49, 50]. In these cells, germline Kv3.1/Kv3.3 deletion predictably broadens action potentials and impairs high‐frequency firing [51]. By contrast, our recordings were obtained from regular‐spiking glutamatergic pyramidal neurons in the aIC. Although the detailed mechanisms underlying this discrepancy remain to be further explored, differences in species, brain region, cell type and neuronal compartment are likely to contribute to this discrepancy.

To our knowledge, this is the first study to triangulate human imaging signatures of post‐TBI anxiety with single‐cell transcriptomic identity and projection‐specific causality in rodents. The cross‐species preservation of both hyper‐connectivity (rs‐fMRI) and neuronal firing‐coupling (fiber photometry) validates a translatable imaging‐behaviour endophenotype that can be deployed in future clinical trials. By demonstrating that KCNC3 (Kv3.3) downregulation play an important role for hyperactivation of the aIC‐lOFC glutamatergic axis. Our results provide a mechanistic rationale and biological target for the future development or repurposing of KCNC3‐positive modulators as potential first‐in‐class anxiolytics in the context of TBI. Future studies employing selective KCNC3 modulators in vivo will be required. And systematic longitudinal studies, examination of different injury severities and models, and cross‐species validation (including human cohorts) will be required to determine whether KCNC3 downregulation robustly and prospectively predicts anxiety outcomes. These lines of evidence will be critical for establishing KCNC3 as a reliable biomarker for anxiety in patients with TBI.

Several limitations of the present work point to important directions for future research. For example, a key limitation of the present study is that our mechanistic conclusions are derived from experiments performed in male mice. Future work will require adequately powered, sex‐inclusive prospective designs that pair behavioral phenotyping with matched molecular, circuit, and electrophysiological analyses to determine whether the KCNC3–excitability mechanism generalizes across sexes or reflects sex‐specific pathways after TBI. And the mouse model cannot fully replicate the complexity of human TBI, and future research should aim to validate these findings in models that more closely mimic clinical conditions. Additionally, TBI may affect multiple brain and neurotransmitter systems; the aIC‐lOFC circuit and KCNC3 may represent only part of the underlying mechanisms. We acknowledge that the upstream signaling pathways linking traumatic brain injury to reduced KCNC3 expression remain unknown. Our single‐cell transcriptomic data indicate that KCNC3 downregulation reflects decreased transcription, and bioinformatic analysis of the KCNC3 promoter highlights several TBI‐relevant transcription factors (NF‐κB, CREB, REST) as candidate mediators to be tested in future mechanistic studies. The roles of other brain regions and molecular pathways warrant further exploration. The mechanisms conferring resilience to post‐TBI anxiety, such as compensatory synaptic adaptations, differential inflammatory responses, or pre‐existing molecular profiles, remain unknown and represent a key direction for future investigation.

Collectively, our study provides the first comprehensive mechanistic account revealing how TBI triggers anxiety through a cascade initiated by the downregulation of KCNC3 in aIC glutamatergic neurons. By identifying KCNC3 and the aIC‐lOFC circuit as key players in this process, our work establishes a robust theoretical framework and highlights a highly promising target for the development of effective therapeutic interventions aimed at mitigating post‐traumatic anxiety and improving the long‐term outcomes for TBI survivors.

## 4 Experimental Section

### 4.1 Animals

SPF‐grade C57BL/6J male mice (body weight 20–25 g; age: 6–8 weeks) were used in this study. The experimental animals were raised in strict accordance with the feeding standard, and the density of each cage was not more than 5 animals. All mice were housed under stable temperature at 24±1°C and consistent humidity between 40% and 60% with a 12‐h light / dark cycle. All animals had free access to food and water. All procedures were approved by the Animal Care Committee of the Soochow University (Suzhou, Jiangsu, China; Approval number: 202312A0844).

### 4.2 TBI Model

TBI was induced using a digital electromagnetically controlled cortical impact (CCI) device (RWD, China) as described in our previous work [52]. Briefly, mice were first anesthetized with 1.5%–3% isoflurane in a mixture of 30% oxygen and 70% nitrous oxide. They were then placed on a heating pad to maintain their body temperature at 37 ± 0.5°C and secured in a stereotaxic frame. Following skull exposure, a circular craniotomy (2.5 mm in diameter) was performed over the right Frontal area (1 mm posterior to the bregma and 1 mm lateral to the sagittal suture) using a motorized drill. The CCI model was established using a 2 mm flat‐tip impactor, with the following parameters: an impact velocity of 4 m/s, a dwell time of 100 ms, and an impact depth of 0.9 mm. After the procedure, the mice were returned to the heating pad to maintain their body temperature during recovery from anesthesia. Sham‐operated mice underwent the same craniotomy procedure without the CCI application.

### 4.3 Modified Neurological Severity Score Determination

To evaluate the neurological status of mice following traumatic brain injury (TBI), we employed the Modified Neurological Severity Score (mNSS). This scoring system comprehensively assesses various aspects of neurological function, including motor ability, sensory perception, balance, and reflexes [53]. The mNSS encompasses four categories, which together yield a total score ranging from 0 to 14 points. A score of 0 indicates normal neurological function, while a score of 14 indicates the maximum deficit in neurological function. Moderate TBI was defined as NSS ≥5 and ≤ 9 within 24 h post‐TBI [54].

### 4.4 Anxiety Behavior Tests

All mice were systematically acclimated to the testing environment for 30 min per day and to the experimenter through 10 min of gentle handling daily, over a period of seven days preceding the experimental procedures.

### 4.5 Open Field Test

The experimental arena, constructed from opaque PVC and measuring 40 × 40 × 40 cm, was meticulously calibrated using Anymaze software (version 7.35, Stoelting Co., USA). Calibration parameters were precisely defined, including a scale of 1 cm equating to 15 pixels, a designated central zone measuring 20 × 20 cm, and test durations set at either 5 or 10 min. The software recorded and analyzed the mice's movement trajectories, including the distance and the time spent in the central area [55, 56]. All behavioral recordings were performed in 10‐min sessions, with each mouse tested only once per batch. For optogenetic modulation experiments, mice underwent two 5‐min recording sessions (light‐off and light‐on conditions). In both optogenetic and chemogenetic experiments, mice were recorded under three conditions (light‐off, light‐on, and light‐on with hM3Dq/hM4Di activation), with each condition lasting 5 min. During the optogenetic experiments, mice were connected to the apparatus and allowed a 5‐min recovery period before being placed in a corner of the open field apparatus for behavioral testing. Each OFT session comprised two alternating 5‐min epochs: one with laser stimulation off and one with the stimulation on. The time and distance each mouse spent in the central area of the field was monitored and recorded using Anymaze software. Comparisons were made for each mouse regarding the time and distance spent in the central area before and after light stimulation [57].

### 4.6 Elevated Plus Maze

The elevated plus maze (EPM) consists of two open arms (30 × 5 cm, no walls), two enclosed arms (30 × 15 × 5 cm, surrounded by three walls), and a central platform (5 × 5 cm), elevated 75 cm above the floor. Each mouse was placed in the center facing an enclosed arm and allowed to explore for 5 min utilizing different batches of mice, and the distance and the time spent in the open arm was reworded using the Anymaze software [58, 59]. For optogenetic modulation experiments, mice underwent two 5‐min recording sessions (light‐off and light‐on conditions). In both optogenetic and chemogenetic experiments, mice were recorded under three conditions (light‐off, light‐on, and light‐on with hM3Dq/hM4Di activation), with each condition lasting 5 min. Prior to optogenetic testing, mice were connected to the experimental setup and allowed 5 min for habituation before behavioral assessment. EPM consisted of alternating 5‐min epochs (light‐off and light‐on stimulation). For each mouse, the time and distance spent in the open arms were compared between pre‐ and post‐stimulation periods [57].

To assess anxiety‐like behaviors in Sham, non‐anxious, and anxious mice, separate cohorts were used for OFT and EPM tests at distinct time points, preventing repeated exposure to the same apparatus. In optogenetics and chemogenetics experiments, the EGFP‐expressing group served as the negative control to account for potential confounds from repeated testing.

### 4.7 Stereotaxic Brain Surgery

Mice were anesthetized via intraperitoneal injection of pentobarbital (dose: 83 mg/kg body weight) and secured in a stereotaxic frame (RWD, 71000‐M, Shenzhen, China) to maintain a horizontal position. Target brain regions were identified based on Bregma coordinates using the third edition of the mouse brain atlas by Keith B.J. Franklin and George Paxinos: the coordinates for the lOFC were set at (AP: +2.5 mm; ML: +1.0 mm; DV: ‐2.7 mm), and for the aIC at (AP: +0.98 mm; ML: +3.50 mm; DV: ‐3.80 mm). A 10 µL microsyringe (Hamilton, P/N: 7653‐01) was used to withdraw the viral solution, which was then injected at a constant rate of 15 nL/min using a microinjection pump (RWD, KDS LEGATO 130), with a total volume of 150 nL. The injector was held in place for 10 min post‐injection to allow for viral diffusion, after which it was slowly removed over approximately 1 min. Mice were returned to their cages and allowed to recover for three weeks prior to further experimental testing.

### 4.8 Fiber Photometry Recording

To assess the baseline concentration of intracellular calcium ions, we conducted calcium imaging experiments. A total of 150 nL of AAV2/9‐vglut2‐GCaMP6f‐EGFP (from BrainVTA, Wuhan, 2.94 × 10^12^ genome copies/mL) was injected into the lOFC at coordinates (AP: +2.5 mm; ML: +1.0 mm; DV: ‐2.7 mm) and the aIC at (AP: +0.98 mm; ML: +3.50 mm; DV: ‐3.80 mm).

For recording calcium signals from axons of aIC^KCNC3+^ glutamate neurons projecting to lOFC in anxious TBI mice, mixed AAV2/9‐vglut2‐Cre (from BrainVTA, Wuhan, 5.07 × 10^12^ genomic copies/mL), AAV2/9‐GCaMP6f‐DIO (from BrainVTA, Wuhan, 5.43 × 10^12^ genomic copies/mL) and AAV2/9‐CMV‐DIO‐KCNC3‐3XFLGA‐P2A‐mCherry (from BrainVTA, Wuhan, 5.0 × 10^12^ genomic copies/mL) or AAV2/9‐CMV‐DIO‐3XFLGA‐P2A‐mCherry (from BrainVTA, Wuhan, 5.0 × 10^12^ genomic copies/mL) were injected into the aIC, prior to implantation of the optical fiber in the lOFC.

For recording calcium signals from axons of aIC^KCNC3+^ glutamate neurons projecting to lOFC in non‐anxious TBI and Sham mice, mixed AAV2/9‐vglut2‐Cre (from BrainVTA, Wuhan, 5.07 × 10^12^ genomic copies/mL), AAV2/9‐GCaMP6f‐DIO (from BrainVTA, Wuhan, 5.43 × 10^12^ genomic copies/mL) and AAV2/9‐CMV‐DIO‐KCNC3‐shRNA‐mCherry or AAV2/9‐CMV‐DIO‐shRNA‐mCherry (from BrainVTA, Wuhan, 5.0 × 10^12^ genomic copies/mL) were injected into the aIC, prior to implantation of the optical fiber in the lOFC.

For recording glutamate release signals in lOFC during EPM stimulation, AAV2/9‐hySn‐iGluSnFR‐WPRE‐PA (from BrainVTA, Wuhan, 5.25 × 10^12^ genomic copies/mL) was injected unilaterally into the lOFC prior to implantation of the optical fiber in the lOFC.

For recording glutamate signals from aIC^KCNC3+^ glutamate neurons projecting to lOFC in anxious TBI mice, mixed AAV2/9‐iGluSnFR (A184S) (from BrainVTA, Wuhan, 5.25 × 10^12^ genomic copies/mL) and AAV2/R‐vglut2‐Cre (from BrainVTA, Wuhan, 5.60 × 10^12^ genomic copies/mL) were injected in lOFC, AAV2/9‐CMV‐DIO‐KCNC3‐3XFLGA‐P2A‐mCherry or AAV2/9‐CMV‐DIO‐3XFLGA‐P2A‐mCherry (from BrainVTA, Wuhan, 5.0 × 10^12^ genomic copies/mL) and AAV2/9‐DIO‐hM3Dq‐EGFP (from BrainVTA, Wuhan, 5.11 × 10^12^ genomic copies/mL) were injected into the aIC, prior to implantation of the optical fiber in the lOFC.

For recording glutamate signals from axons of aIC^KCNC3+^ glutamate neurons projecting to lOFC in non‐anxious TBI and Sham mice, mixed AAV2/9‐iGluSnFR (A184S) (from BrainVTA, Wuhan, 5.25 × 10^12^ genomic copies/mL) and AAV2/R‐vglut2‐Cre (from BrainVTA, Wuhan, 5.60 × 10^12^ genomic copies/mL) were injected in lOFC, AAV2/9‐CMV‐DIO‐KCNC3‐3XFLGA‐P2A‐mCherry (from BrainVTA, Wuhan, 5.0 × 10^12^ genomic copies/mL) and AAV2/9‐DIO‐hM3Dq‐EGFP (from BrainVTA, Wuhan, 5.11 × 10^12^ genomic copies/mL)were injected into the aIC, prior to implantation of the optical fiber in the lOFC. Low‐fluorescence multimode fibers (Doric, MF1.25, MFP_200/220/9000.37_2mFCM_MF1.25_LAF) were implanted into the target brain regions. Mice were allowed a three‐week recovery period, followed by a three‐day acclimation to the experimental environment prior to subsequent experiments. The implanted ceramic fiber connectors were linked to a switch using fiber jumpers (R‐FC‐L‐N3‐200‐L1, 2 m length, compatible with 1.25 mm ceramics). Fluorescent signals were excited using a 470 nm laser, and calcium signals were subsequently captured using an in vivo calcium imaging system. Data analysis was performed with recording software (RWD Life Science, Shenzhen, China) and further processed in MATLAB. The formula for calculating fluorescence changes was ΔF/F = (F—F1) / F0, where F0 represents the baseline photometric signal, and F1 represents the baseline‐corrected photometric value [60, 61].

### 4.9 Optogenetics Manipulation

Optogenetics allows for the precise modulation of neuronal populations through the introduction of light‐sensitive proteins via adeno‐associated virus vectors. In this study, we utilized this technology, along with specific viral tools and transgenic animal models, to target the glutamatergic circuitry between the aIC and the lOFC. We performed unilateral injections of AAV2/9‐vglut2‐hChR2‐EGFP or AAV2/9‐vglut2‐EGFP (from BrainVTA, Wuhan, 6.27 × 10^12^ genomic copies/mL) and AAV2/9‐vglut2‐eNpHR‐EGFP or AAV2/9‐vglut2‐EGFP (from BrainVTA, Wuhan, 6.27 × 10^12^ genomic copies/mL) to achieve selective optical activation or inhibition of glutamatergic neurons in the aIC and lOFC. Low‐fluorescence multimode fibers (Doric, MF1.25, MFP_200/220/9000.37_2mFCM_MF1.25_LAF) were unilaterally implanted in the OFC or IC, positioned 100–200 µm above the injection site. Three weeks post‐injection (with mice allowed at least one week of recovery and a 30–60 min acclimation period prior to behavioral testing), the mice underwent optogenetic stimulation. Laser stimulation was applied at either 473 or 589 nm, controlled by an optogenetic system (RWD Life Science, Shenzhen, China), with parameters set for each opsin. All procedures were conducted in mice expressing EGFP.

### 4.10 Chemogenetic Manipulation

For chemogenetic manipulation of the aIC‐lOFC pathway, AAV2/9‐vglut2‐hM3Dq‐mCherry (from BrainVTA, Wuhan, 5.0 × 10^12^ genomic copies/mL), AAV2/9‐vglut2‐hM4Di‐mCherry (from BrainVTA, Wuhan, 5.13 × 10^12^ genomic copies/mL) AAV2/9‐DIO‐hM3Dq‐EGFP (from BrainVTA, Wuhan, 5.11 × 10^12^ genomic copies/mL) and AAV2/9‐DIO‐hM4Di‐EGFP (from BrainVTA, Wuhan, 5.13 × 10^12^ genomic copies/mL) injected bilaterally into the lOFC were used for projection‐specific activa‐tion or inhibition.

### 4.11 Drug Administration

For behavioral experiments, D‐AP5 (30 mm, 1 µL) was stereotactically injected into the left side of the lOFC of anxious TBI mice [62]. Saline or CNO (300 nL per side) was microinjected bilaterally via infusion cannulas into the lOFC or aIC 30 min before the EPM behavior test at a flow rate of 100 nL/min. The injector cannulas were linked to 10 mL microsyringes (Gaoge, Shanghai, P.R. China) through polyethylene tubing and were positioned on a microsyringe pump (KD Scientific, LEGATO130, USA) [63]. The injecto rcannulas were kept in the lOFC or aIC for an additional 2–3 min after the injection to allow for adequate local drug diffusion.

### 4.12 Virus Tracing

For retrograde tracing, AAV2/R‐vglut2‐EGFP (from Wuhan BrainVTA, 5.56 × 10^12^ genome copies/mL) was injected into the lOFC. Anterograde tracing was performed using AAV2/9‐vglut2‐EGFP (from Wuhan BrainVTA, titer: 6.27 × 10^12^ genome copies/mL). For monosynaptic anterograde tracing, AAV2/1‐vglut2‐Cre (from Wuhan BrainVTA, 1.05 × 10^13^ genome copies/mL) was injected into the aIC, and AAV2/9‐EF1α‐DIO‐mCherry (from Wuhan BrainVTA, 5.24 × 10^12^ genome copies/mL) was injected into the lOFC.

### 4.13 Single‐Cell Transcriptomics

Fresh tissues were collected, minced on ice, snap‐frozen in liquid nitrogen, and stored at −80°C or in liquid nitrogen. Matched pieces were used to assess RNA integrity before large‐scale processing. For single‐nucleus RNA sequencing, nuclei were isolated from frozen tissues by Dounce homogenization in sucrose‐based lysis buffer under RNase‐free, ice‐cold conditions, followed by filtration, iodixanol density gradient centrifugation, and quality control; only high‐quality nuclear suspensions were used for library preparation. Single‐nucleus libraries were prepared with the Chromium Next GEM Single Cell 3′ Reagent Kits v3.1 and sequenced on an Illumina NovaSeq X Plus. Raw data were processed with Cell Ranger (v6.1.0) for sample demultiplexing, barcode processing, 3′ gene counting, FASTQ generation, alignment to the Ensembl release 110 reference genome using STAR, and construction of gene–barcode matrices after UMI collapsing and removal of low‐confidence and duplicate reads. Gene‐cell‐barcode matrices from individual libraries were aggregated with read‐depth normalization. Low‐quality nuclei and multiplets were removed using DoubletFinder and Seurat, retaining cells with 380–5500 detected genes, ≤25,000 UMIs, and ≤10% mitochondrial transcripts. To correct batch effects, all samples were integrated with Harmony using PCA embeddings and batch assignments to obtain a batch‐corrected embedding. Seurat performed graph‐based clustering by constructing a shared‐nearest neighbor (SNN) graph in PCA space and applying the Louvain algorithm. Clusters were visualized with t‐distributed stochastic neighbor embedding (t‐SNE) and annotated based on classic marker genes. Differential expression for each cluster vs. all other cells was tested with a likelihood‐ratio test, and differentially expressed genes were defined by P ≤ 0.01, log2(fold change) ≥ 0.36067 [64, 65]; and detection in >25% of cells in the cluster. All genes were mapped to GO terms in the GO database (http://www.geneontology.org/). GO enrichment analysis was performed by mapping genes to GO terms, testing enrichment with a hypergeometric test against the genome background, and correcting *p* values by false discovery rate (FDR)‐corrected, with FDR ≤ 0.05 defining significantly enriched GO terms [65, 66].

### 4.14 Whole‐Cell Recordings

The aIC of anxious TBI mice were injected with AAV2/9‐vglut2‐cre and AAV2/9‐CMV‐DIO‐KCNC3‐3XFLGA‐P2A‐mCherry or AAV2/9‐CMV‐DIO‐3XFLGA‐P2A‐mCherry. Non‐anxious TBI and Sham mice were injected with AAV2/9‐vglut2‐cre and AAV2/9‐CMV‐DIO‐KCNC3‐shRNA‐mCherry or AAV2/9‐CMV‐DIO‐shRNA‐mCherry. After 21 days of viral expression, mice were deeply anesthetized with 1% sodium pentobarbital (100 mg/kg, intraperitoneally) and then perfused with 30 mL rapidly ice‐oxygenated dissection buffer that contained: sucrose (210 mm), sodium pyruvate (3.1 mm), sodium l‐ascorbate (11.6 mm), NaH_2_PO_4_(1.0 mm), NaHCO_3_ (26.2 mm), MgCl_2_ (5.0 mm) and glucose (20.0 mm) at pH 7.4.

Brains were quickly removed and placed in ice‐cold, oxygenated artificial cerebrospinal fluid (aCSF; continuously bubbled with 95% O_2_/5% CO_2_), containing: 124 mm NaCl, 2.5 mm KCl, 2 mm MgSO_4_, 2.5 mm CaCl_2_, 1.25 mm NaH_2_PO_4_, 22 mm NaHCO_3_, and 10 mm glucose. Coronal brain slices (300 µm thickness) containing the anterior insular cortex (aIC) were prepared using a vibrating microtome (VT1200S, Leica). Slices were transferred to a holding chamber containing oxygenated aCSF at 32°C–34°C for an initial recovery period (≥30 min), and then maintained at room temperature until recording.

Whole‐cell current‐clamp recordings were obtained from visually identified pyramidal neurons in the aIC using a MultiClamp 700B amplifier (Molecular Devices) mounted on an upright microscope (BX51WI, Olympus). During recording, slices were continuously perfused with oxygenated aCSF at a constant flow rate using a peristaltic pump, and the liquid level in the recording chamber was kept stable. Patch pipettes were pulled from borosilicate glass capillaries using a horizontal puller (Sutter P‐1000) to a resistance of 4–6 mΩ when filled with internal solution. The internal pipette solution contained (in mM): KCl 140, MgCl_2_ 1, CaCl_2_ 0.5, EGTA 5, HEPES 10, and ATP 3 (pH 7.4 with KOH). Pipettes were filled with a standard intracellular solution and mounted on the headstage. Under low magnification, the pipette tip was positioned in the aIC, and under higher magnification, the pipette was advanced to contact visually healthy neurons with smooth somatic membranes. A high‐resistance seal was formed, followed by brief suction to achieve whole‐cell configuration. Series resistance and basic membrane properties were continuously monitored, and only neurons with stable access and membrane parameters throughout the recording were included in the analysis. Data were acquired using a Digidata 1550A digitizer (Molecular Devices) and Clampex 10 software (Molecular Devices). After membrane rupture and stabilization of the resting membrane potential (RMP), cells were held at their natural RMP and subjected to a series of depolarizing current injections using predefined current‐clamp protocols to evoke action potentials (APs).

### 4.15 People MRI Data Acquisition and Processing

Patients included in this study were admitted to the Department of Neurosurgery at Zhangjiagang Hospital, affiliated with Soochow University. The study protocol was approved by the Research Ethics Committee of the Affiliated Zhangjiagang Hospital of Soochow University (reference number: ZJGYYLL2023‐10‐047). Informed consent for functional MRI BOLD imaging was obtained from all participants, who were capable of cooperating well with the procedure. This study was approved by the ethics committee. These patients were suspected of having traumatic anxiety based on detailed telephonic interviews, physical examinations, electroencephalograms, and cranial MRIs. The control group consisted of 15 non‐anxiety TBI patients, matched for age and gender with 15 anxiety TBI patients in the experimental group.

These patients, aged 18–50 years, showed no abnormalities on routine cranial MRI scans conducted 1–2 years post‐discharge. All patients had moderate traumatic brain injury, with lesion heterogeneity controlled by restricting the injury site to frontal lobe damage. Participants had no severe physical diseases, no substance or alcohol dependence, and no other conditions that could affect brain structure and function. They also had no family history of neurological diseases or mental disorders. In the experimental group, anxiety symptoms were confirmed by a Hamilton Anxiety Rating Scale (HAMA) score greater than 14, whereas patients in the control group had HAMA scores lower than 14. None of the participants were taking psychotropic medications or other drugs that might influence brain function at the time of the study. Sample characteristics are shown in Table 1. Given observed differences in age, sex, and educaton level, these variables were included as covariates in the fMRI analysis. All participants were informed of the study's content both orally and in writing and provided written informed consent to undergo BOLD‐fMRI.All participants underwent scanning using a Philips Achieva 3.0 T superconducting MRI scanner with a standard head coil at Zhangjiagang Hospital, affiliated with Soochow University. Scans were performed by trained technicians at this facility.

### 4.16 Functional MRI Scanning Parameters

Gradient Echo (GE) Echo‐Planar Imaging (EPI) Parameters for Resting‐State Acquisition: Field of View (FOV): 224 × 239 mm^2^; Matrix Size (MS): 112 × 116; Repetition Time (TR): 3000 ms; Echo Time (TE): 30 ms; Number of Averages (NA): 1; Number of Repetitions (NR): 184; Spatial Resolution: 2 × 2 × 3 mm^3^; Slices: 45, no gap; Scan Duration: 9 min and 12 s; Sagittal T1W_3D_TFE Gradient Echo Sequence Parameters:FOV: 250 × 250 mm^2^; MS: 228 × 227; TR: 3000 ms; TE: 48.72 ms; NA: 1; NR: 1; Spatial Resolution: 1 × 1 × 1.2 mm^3^; Slices: 301, no gap; Scan Duration: 4 min and 59 s.

### 4.17 Functional MRI Data Analysis

1) Degree centrality (DC) describes the number of short‐distance connections for each node. Nodes with higher centrality contribute more significantly to the overall network efficiency. Higher node degrees indicate higher DC values, suggesting the node's importance within the network. We first calculated the correlation strength between each voxel and all other voxels in the brain, using a threshold of 0.2 to exclude weak connections. The weighted average of the remaining voxel correlation strengths was taken as the DC value for statistical analysis. To identify TBI‐induced anxiety‐related alterations in DC, whole‐brain voxel‐wise two‐sample *t*‐tests were performed. In line with widely used recommendations for cluster‐wise inference in fMRI, we adopted a voxel‐level threshold of p < 0.001 (uncorrected) combined with a cluster‐level threshold of p < 0.05, family‐wise error (FWE)‐corrected. This combination is considered relatively conservative while maintaining adequate power in datasets of this size, and has been extensively used in both human and preclinical fMRI studies [33, 67]. These thresholds and analysis settings were pre‐specified before examining the statistical maps and were not adjusted post hoc to obtain significance.

2) Whole‐Brain functional connectivity matrix analysis: Based on the human brain functional atlas, we analyzed and extracted the mean time series of each region of interest (ROI). We calculated the connection strength between each pair of ROIs (ROI‐ROI) and applied Fisher z‐transformation to the connectivity matrices. Two‐sample *t*‐tests with voxel‐level *p* < 0.001, cluster‐level *p* < 0.05, FWE‐corrected were conducted to analyze changes in ROI‐ROI connectivity between the Sham group and TBI‐induced anxiety and depression groups. This thresholding scheme is recommended in many fMRI studies to reduce false positives compared to more liberal voxel‐wise thresholds (*p* < 0.01 or *p* < 0.05) while maintaining adequate power to detect biologically meaningful clusters, particularly in preclinical datasets with limited sample sizes.

3) Seed‐based functional connectivity analysis: Key ROIs were selected as seed points (related to anxiety and depression regions). The average time series of voxels within each ROI was extracted, and correlations between the time series of the seed ROI and all other brain voxels were computed. Fisher z‐transformation was applied, followed by voxel‐wise two‐sample *t*‐tests with threshold at voxel *p* < 0.001, cluster *p* < 0.05, FWE‐corrected to determine functional connectivity differences between TBI patients and normal Sham controls.

### 4.18 Animal MRI Data Acquisition and Processing

Experimental mice were initially placed in an induction anesthesia chamber and anesthetized with isoflurane. They were then transferred to the uMR 9.4T (United Imaging Life Science Instrument, Wuhan, China) MRI animal bed, where they were secured using bilateral ear bars and a frontal tooth bar. Isoflurane dosage was gradually reduced to approximately 1.5%–3% to maintain the mice in a light, stable anesthetic state, with a respiratory rate of about 60–80 breaths per minute. Throughout the experiment, a custom‐made warm water circulation system was used to cover the mice and maintain their body temperature [68, 69].

### 4.19 Functional MRI Scanning Parameters

Coronal Gradient Echo (GE) Echo‐Planar Imaging (EPI) Parameters for Resting‐State Acquisition:Field of View (FOV): 21.0 × 28.0 mm^2^; Matrix Size (MS): 80 × 60; Repetition Time (TR): 2000 ms; Echo Time (TE): 14 ms; Number of Averages (NA): 1; Number of Repetitions (NR): 300; Spatial Resolution: 0.35 × 0.35 × 0.8 mm^3^; Slices: 22, no gap; Scan Duration: 10 min; Coronal T2 Structural Image Turbo‐RARE Sequence Parameters: FOV: 21.0 × 28.0 mm^2^; MS: 256 × 256; TR: 3000 ms; TE: 12 ms; NA: 4; NR: 1; Spatial Resolution: 0.08 × 0.11 × 0.8 mm^3^; Slices: 22, no gap; Scan Duration: 6 min 24 s.

### 4.20 Functional MRI Data Analysis

Degree centrality describes the number of short‐distance connections for each node. Nodes with higher centrality contribute more significantly to the overall network efficiency. Higher node degrees indicate higher DC values, suggesting the node's importance within the network. We first calculated the correlation strength between each voxel and all other voxels in the brain, using a threshold of 0.2 to exclude weak connections. The weighted average of the remaining voxel correlation strengths was taken as the DC value for statistical analysis. Whole‐brain voxel‐wise two‐sample *t*‐tests with voxel‐level *p* < 0.001, cluster‐level *p* < 0.05, FWE‐corrected. were then used to obtain difference images for TBI‐induced anxiety.

### 4.21 Real‐Time Quantitative Polymerase Chain Reaction

Real‐time quantitative polymerase chain reaction (RT‐qPCR) was utilized to assess the mRNA expression levels in aIC of mice subjected to Sham surgery and TBI. Initially, aIC tissue was harvested and placed in 1.5 mL centrifuge tubes, followed by the addition of Trizol (TransGen Biotech) to ensure complete tissue lysis. Subsequently, chloroform (in a 5:1 ratio with Trizol) was added, and the mixture was vortexed for 15 s before being allowed to stand at room temperature for 2 min. The samples were then centrifuged at 12 000 g for 10 min at 4°C. The upper aqueous phase, containing RNA, was carefully transferred to a new tube and precipitated with an equal volume of isopropanol. After centrifugation at 8,000 g for 10 min at 4°C, the ethanol was removed, and the RNA pellet was air‐dried. The RNA was then reconstituted in 20 µl of RNase‐free dH_2_O, and its concentration was quantified using a Nanodrop spectrophotometer. For the reverse transcription reaction, reagents were prepared according to the protocol provided by the reverse transcription kit (TransGen Biotech). The reverse transcription was conducted in a PCR instrument (Bio‐Rad) under the following conditions: 85°C for 5 s, followed by 42°C for 15 min, and concluded at 4°C. This procedure converted the mRNA from the aIC into complementary cDNA.Subsequently, primers (Table 2), components of PerfectStart Green qPCR SuperMix (+Dye II), and the synthesized cDNA were combined in a centrifuge tube and subjected to qPCR using an ABI 7500 instrument (USA). The qPCR cycling conditions were as follows: initial denaturation at 95°C for 10 min, followed by 40 cycles of denaturation at 95°C for 15 s and annealing/extension at 60°C for 45 s. After the qPCR run, the cycle threshold (Ct) values were recorded, and quantitative analysis was performed using the comparative Ct method [70, 71, 72].

### 4.22 Western Blotting

Tissue samples from the aIC were collected and immediately homogenized in centrifuge tubes containing RIPA lysis buffer (Biyuntian, catalog no. P0013B) supplemented with a phosphatase inhibitor mixture (Biyuntian) using an ultrasonic disruptor. The homogenate was centrifuged at 12 000 × g for 10 min at 4°C to obtain the supernatant. Protein concentrations were quantified using a BCA protein assay kit (Thermo), following the manufacturer's instructions. A total of 30 µg of protein from each sample was diluted in an equal volume of physiological saline and subjected to electrophoresis on a 10% sodium dodecyl sulfate‐polyacrylamide gel (Bio‐Rad). After electrophoresis, proteins were transferred to a polyvinylidene fluoride (PVDF) membrane (Merck Millipore) using a fast transfer buffer (Newcell) at a constant current of 400 mA for 40 min at 4°C. Following transfer, the PVDF membrane was blocked at room temperature for 2 h with 5% non‐fat dry milk in TBST. The membrane was then incubated overnight at 4°C with primary antibodies: anti‐GAPDH (1:1000, GoHere Technology, AB‐P‐R 001) and anti‐KCNC3 (1:1000, Abcam, AB‐166608). After primary antibody incubation, the membrane was washed three times with TBST for 10 min each. Subsequently, the membrane was incubated with appropriate secondary antibodies at room temperature for 2 h, followed by three additional washes with TBST, each lasting 10 min. Signal detection was performed using an enhanced HRP chemiluminescence detection kit (EZ‐ECL, Biological Industries, catalog no. 20‐500‐120) and a chemiluminescence imaging system (ChemiDoc XRS, Bio‐Rad, Hercules, CA, USA). Band densities were quantified using ImageJ software, and protein expression levels were normalized to GAPDH to account for loading variations.

### 4.23 Immunofluorescence Staining

Mice were deeply anesthetized and transcardially perfused with 0.9% normal saline, followed by 4% paraformaldehyde in phosphate‐buffered saline (PBS, pH 7.2–7.4). Brains were removed, post‐fixed at 4°C (either overnight or for at least 2 h), and cryoprotected sequentially in 10%, 20%, and 30% sucrose solutions. Coronal brain sections (25 µm thick) were prepared using a freezing microtome (CM3050 S, Leica, Germany) and either processed immediately or stored at −20°C. For staining, sections were equilibrated to room temperature and subjected to antigen retrieval in boiling sodium citrate buffer for 10 min. After washing three times in PBS (10 min each), sections were blocked for 1 h at room temperature in PBS containing 7% normal donkey serum, 0.3% Triton X‐100, and 0.05% sodium azide. Sections were then incubated overnight at 4°C with primary antibodies: rabbit anti‐Glutamate (1:200, Sigma–Aldrich, USA), rabbit anti‐GABA (1:200, Sigma–Aldrich, USA), mouse anti‐c‐Fos (1:200, asis Biofarm, China), mouse anti‐KCNC3 (1:20, Santa Cruz Biotechnology, USA). After primary antibody incubation, sections were washed again three times for 10 min each in PBS and incubated with a fluorescent dye‐conjugated secondary antibody for 1 h at room temperature. Following three washes (10 min per time) with PBS, the brain sections were incubated with DAPI. The secondary antibody included anti‐rabbit Alexa Fluor 488 (1:750, Invitrogen, USA) and anti‐rabbit Alexa Fluor 555 (1:750, Invitrogen, USA).

### 4.24 Statistical Analysis

Statistical analyses were conducted using GraphPad Prism version 8.0. Data are expressed as mean ± standard error of the mean (SEM). Outlier data points were identified as those deviating from the mean by more than 0.2 standard deviations. One‐way ANOVA with Tukey's post hoc test was used for single‐factor designs with ≥3 groups; two‐way ANOVA with Sidak's correction was applied to factorial designs with pre‐specified contrasts; two‐tailed unpaired *t*‐test were restricted to two‐group comparisons under approximate normality and homoscedasticity; and Wilcoxon tests were used when normality assumptions were not met or sample sizes were small. Multiple‐comparison corrections (Tukey/Sidak/FWE) were systematically applied whenever more than one comparison was made within a family of hypotheses. A p‐value of less than 0.05 was deemed statistically significant.

## Author Contributions

M.‐G.L., X.‐B.Q. and H.‐L.Z. performed experiments, analyzed data and prepared the manuscript. D.L., L.J. and X.‐C.X. performed experiments, analyzed data, and prepared the figures. J.‐S.D. and L.‐J.Y. analyzed data and prepared the manuscript. R.G., G.‐Y.X. and L.Z. designed experiments, supervised the experiments, and finalized the manuscript. All the authors have read and approved the paper.

## Ethics

Care and handling of the animals were approved by the Institutional Animal Care and Use Committee of Soochow University Ethics Committee's guidelines (Suzhou, jiangsu, China; Approval number: 202312A0844). Patients induced in this study were approved by the Research Ethics Committee of the Affiliated Zhangjiagang Hospital of Soochow University (Reference number: ZJGYYLL2023‐10‐047).

## Conflicts of Interest

The authors declare no conflicts of interest.

5

**TABLE 1:** Demographic data and clinical characteristics of subjects.

	non‐anxious TBI n = 15	anxious TBI n = 15	*P* value
Age,y	41.07±5.667	44.07±7.357	0.4791
Sex,M/F	7/8	9/6	0.9699
Education,y	13±4.069	11±2.869	0.725

**TABLE 2:** Primer sequences used in the present study.

Primers Sequence (5' to 3')	
C2cd2l‐F	GGAACTGATCAAGGACGCTATA
C2cd2l‐R	GAAGCTGCCGTAAGAATAATCG
Fndc5‐F	GCGGGTAGCACAGGAGAGGAG
Fndc5‐R	CGTTCAGCAGGGATGGAAGTCAC
Calr‐F	GTCGAATCCAAACATAAGTCCG
Calr‐R	CGATATTCTGCTCATGCTTCAC
Pebp1‐F	TGGTCAACATGAAGGGTAATGA
Pebp1‐R	CCAGGTTATACTTCTTGCGGAA
Clcn3‐F	CATGGGCAGAGTTAATCATTGG
Clcn3‐R	GAAACTGCAAGAAAGGCAAAAC
Clcn4‐F	CTCAGCTCTGTGACTACATCAA
Clcn4‐R	GAAGAGACCTGAGGGAATCTTC
Kcnma1‐F	TGTTCAGTTTTTGGACCAAGAC
Kcnma1‐R	ATTGTCATTGAAGTATGTCGCG
Slc12a6‐F	GCCTTCTTGGTCCTCATGGTCTTG
Slc12a6‐R	CGGGAAGTGTGGTGGAGCAAAG
Scn3b‐F	CTATTCACCAACCACCCACCCAAC
Scn3b‐R	GGCTCTTCAGTTCAGGCAAGGTC
Kcnc1‐F	ACAGCCACTTCGACTATGAC
Kcnc1‐R	AATAGTTCAGGATGTGAGCGAA
Kcna1‐F	TCAGTTGCTCCATGTTAGTTCT
Kcna1‐R	CTGTCTGTAATGGGCTATGCTA
Kcnj4‐F	CCTTCTGCTATGAGAATGAGCT
Kcnj4‐R	AAACTCAAGCATCCGGATAATG
Scn2b‐F	GCCACTGCTTCCACAAGAGACTC
Scn2b‐R	TCCTTACTCCATGCCCAACCTACC
Trpm4‐F	GACGTTCATAGTGGACCTCTC
Trpm4‐R	GTTGATCAGCATGTCTCTGTTC
Grik1‐F	CTGGACACTCCTCTATTTCCTG
Grik1‐R	GTGACTGCAAACTTGAAAGCTA
Cacng3‐F	CCTGAAGAAGTCTACATTTGCG
Cacng3‐R	GTGGAACTGTAGAAAAGCATGG
Kcnf1‐F	CTGGGTGTTGACGCTGCTGAG
Kcnf1‐R	TGACCACGGAGGAGACAAGGATG
Cacnb2‐F	CTCCTCCTTATGATGTGGTACC
Cacnb2‐R	TGACTCTTGTGATGGATATCCG
Cnr1‐F	GCTTGATGTGGACTATTGCAAT
Cnr1‐R	TCCAGAACATCAGGTAGGTTTC
Trpc6‐F	CTTCCTGGCTCTCATATACTGG
Trpc6‐R	TGTGCTACAAACTTCATGAACG
Gnal‐F	CTAAAGAGGCGAGGAAAGTCAG
Gnal‐R	GGATCCTCATCTGTTTGACGAT
Gpr85‐F	CTGTGATCTGCATGGTGTGGACTC
Gpr85‐R	GAAGGAGCGGTGTTGGAAGGTG
Gpr88‐F	CTCTGCGAGGAAGAAGAATCG
Gpr88‐R	CGAGATAGATGACCATACCGTT
Kcnc3‐F	CTGCTGCTGGATGACCTATC
Kcnc3‐R	CTGAAAACACAGACGCTTGAG
Grina‐F	GCATGATAGCCAGCTTTTACAA
Grina‐R	GTATACAATCTCCAGGATGCGG
Gpr22‐F	TATTCCACCCTCTCTTGTATGC
Gpr22‐R	CCATGAGTTGTGTATTACAGCG
Paqr9‐F	TTCCACATCTTCACTTTCCTCA
Paqr9‐R	GAACTTCCTGATGACTAGTCCC
Htr4‐F	GATGCTAATGTGAGTTCCAACG
Htr4‐R	AGGCGAGAGACACAATGAAATA
Cachd1‐F	CATGATGACGACTTGGACCTGGAC
Cachd1‐R	AGGTGGTGGCTGTGATGGAGAG

## Supporting information




**Supporting File**: advs76779‐sup‐0001‐SuppMat.docx.

## Data Availability

The raw data from behavioral experiments, Fiber recordings, preprocessed datasets, and behavior from imaging experiments. All data needed to evaluate the conclusions in the paper are present in the paper and/or the Supplementary Materials.
