# Supplementary material for: Targeting the KCNC3‐Dependent Anterior Insular‐Lateral Orbitofrontal Cortex Glutamatergic Circuit Attenuates Post‐Traumatic Anxiety
Source: Adv Sci (Weinh). 2026 Jul 27;13(58):e76779. doi: 10.1002/advs.76779 (PMC13403725; doi:10.1002/advs.76779)
Supplement: Supplementary file 1 [file ADVS-13-e76779-s001.docx]

Supporting Information

**Targeting the KCNC3-Dependent anterior Insula-lateral Orbitofrontal Cortex Glutamatergic Circuit Attenuates Post-Traumatic Anxiety**

Meng-Ge Li^1,#^, Xiao-Bo Qian^1,#^, Hai-Long Zhang^3,#^,Di Li^4^, Ling Ji^1^, Xin-Chun Xu^1^, Jia-Sheng Ding^1^, Li-Jun Yin^1^, Li Zhang^1,*^, Guang-Yin Xu^2,*^, Rong Gao^1,*^

Supplementary Information for

**Targeting the KCNC3-Dependent anterior Insula-lateral Orbitofrontal Cortex Glutamatergic Circuit Attenuates Post-Traumatic Anxiety**

Meng-Ge Li^1,#^, Xiao-Bo Qian^1,#^, Hai-Long Zhang^3,#^,Di Li^4^, Ling Ji^1^, Xin-Chun Xu^1^, Jia-Sheng Ding^1^, Li-Jun Yin^1^, Li Zhang^1,*^, Guang-Yin Xu^2,*^, Rong Gao^1,*^

This file includes:

Figure S1 to S7

**Supplementary Information**

Figure S1. Increased activity and enhanced IC-OFC functional connectivity in TBI patients with anxiety (related to Figure 1).

Figure S2. TBI mice showed moderate injury and no impairment in motor performance (related to Figure 2). A) The modified Neurological Severity Score (mNSS) of TBI mice. B, C) Statistical graph of the total distance in EMP and OFT. Data are presented as mean ± SEM. Statistical analysis was performed using two-tailed unpaired t-test, n = 10 for each group, A; two-way ANOVA followed by Sidak's multiple comparisons test, n = 10 for each group, B, C. ns =not significant, ****p* < 0.001.

Figure S3. Glutamatergic neurons in the aIC projected to glutamatergic neurons in the lOFC (related to Figure 4). A) Representative fluorescence images illustrating the co-localization of glutamatergic neurons and viral cell bodies in the aIC (scale bar, 100 µm). B) Identification of neuron types projecting from aIC to lOFC (scale bar, 50 µm).

Figure S4. EPM stimulation promotes glutamate release in lOFC (related to Figure 5). A) Viral strategy for detecting glutamate levels in lOFC. B, C) Representative heatmap and curve graph of lOFC glutamate levels. D) Bar graph of peak calcium signals and area under the curve during EPM open arm exploration. Data are presented as mean ± SEM. Statistical analysis was performed using one-way ANOVA followed by Tukey's multiple comparisons test, n = 6 per group, D). ns =not significant, ****p* < 0.001.

Figure S5. Cell type–specific marker gene bubble plot and differential expression of KCNC3 across cell populations (related to Figure 6). A) t-distributed Stochastic Neighbor Embedding (t-SNE). B) Cell type–specific marker gene bubble plot**.** C) Volcano plot illustrating distribution of KCNC3 in inhibitory neurons, Astrocyte, microglia and OPC.

Figure S6. Regulation of anxiety behaviors by the aIC^KCNC3+^-lOFC glutamatergic circuit in non-anxious TBI mice (related to Figure 10). A) Viral strategy for detecting glutamate levels in the lOFC with aIC^KCNC3+^-lOFC circuit inhibition in Sham mice. B) Representative heatmap, curve graph of lOFC glutamate levels before and after aIC^KCNC3+^-lOFC circuit inhibition. C) Bar graph of peak calcium signals and AUC of lOFC glutamate levels before and after aIC^KCNC3+^-lOFC circuit inhibition (**p* < 0.5, ***p* < 0.01,). D) Diagram of viral injection sites. E) Representative image of KCNC3 knockdown (scale bar, 50 μm). F, G) Bar graph showing EPM (F) and OFT (G) behavioral outcomes following KCNC3 knockdown in the aIC (**p* < 0.05, ****p* < 0.001, two-tailed unpaired *t*-test and Wilcoxon signed-rank test, n = 6 per group). Data are presented as mean ± SEM. Statistical analysis was performed using two-tailed unpaired *t*-test, n = 6 per group, C, F; two-tailed unpaired *t*-test and Wilcoxon signed-rank test, n = 6 per group, G). **p* < 0.05, ****p* < 0.001.

Figure S7. Regulation of anxiety behaviors by the aIC^KCNC3+^-lOFC glutamatergic circuit in Sham mice (related to Figure 10). A) Viral strategy for detecting glutamate levels in the lOFC with aIC^KCNC3+^-lOFC circuit inhibition in Sham mice. B) Representative heatmap, curve graph of lOFC glutamate levels before and after aIC^KCNC3+^-lOFC circuit inhibition. C) Bar graph of peak calcium signals and AUC of lOFC glutamate levels before and after aIC^KCNC3+^-lOFC circuit inhibition. D) Diagram of viral injection sites. E) Representative image of KCNC3 knockdown (scale bar, 50 μm). F, G) Bar graph showing EPM (F) and OFT (G) behavioral outcomes following KCNC3 knockdown in the aIC. Data are presented as mean ± SEM. Statistical analysis was performed using two-tailed unpaired *t*-test, n = 6 per group, C, F; two-tailed unpaired *t*-test and Wilcoxon signed-rank test, n = 6 per group, G). **p* < 0.05, ***p* < 0.01, ****p* < 0.001.
